# Global models predict clouds at the wrong time of day: Does it matter for radiation and climate?

**DOI:** 10.1126/sciadv.ady3236

**Published:** 2025-12-12

**Authors:** Travis Aerenson, Daniel McCoy, Gregory Elsaesser, Jingbo Wu, Jacqueline M. Nugent, Hunter Brown, August Mikkelsen, Mark D. Zelinka

**Affiliations:** ^1^ University of Wyoming, Laramie, WY 82071, USA.; ^2^ Columbia University, New York, NY 10027, USA.; ^3^ NASA-GISS, New York, NY 10025, USA.; ^4^ Lawrence Livermore National Laboratory, Livermore, CA 94550, USA.

## Abstract

Accurate prediction of future climate change hinges upon the ability of Earth system models (ESMs) to simulate clouds and their radiative effects. Even if an ESM can simulate the correct clouds, a systematic error in the amount of sunlight reflected by clouds (and, thus, cloud radiative effect) can exist if the clouds are simulated at the incorrect time of day. In this work, we develop an analytical model connecting diurnal cloud biases to emergent mean state radiative biases. With the use of satellite observations, we demonstrate that there are errors in the time of day that clouds are occurring in ESMs that would cause bias in shortwave cloud radiative effect (SWCRE) that is greater than 45% of the total SWCRE bias, but such errors in the cloud diurnal cycle are masked by other compensating errors, indicating that these ESMs are getting the right answer for the wrong reasons.

## Teaser

Errors in the energy balance of Earth system models can be traced to the time of day when they simulate clouds.

## INTRODUCTION

Prediction of Earth’s future climate relies upon our ability to predict the absorption of incident solar radiation, the emission of thermal radiation to space, and how they both change because of anthropogenic emissions of heat-trapping gases (such as CO_2_). Perturbations to the global energy balance affect the global mean temperature and local climate (*1*–*4*). Clouds account for large variations in Earth’s albedo, where the amount of sunlight reflected in a particular location depends on the incidence and optical thickness of cloud and coincident sunlight. Clouds affect Earth’s radiation balance through shortwave (SW) and longwave (LW) radiation. They simultaneously reflect sunlight, cooling the surface (often referred to as the SW cloud radiative effect or SWCRE), and inhibit the emission of thermal radiation to space, thus warming the surface (often referred to as the LW cloud radiative effect or LWCRE). Cloud changes in response to changes in global mean temperature can feed back onto the initial radiative forcing imposed onto Earth and can either enhance or dampen the initial warming signal. Predictions of future climate depend upon cloud conditions relative to the timing of incoming solar radiation and how that may change in the future. Here, we discuss how inaccurate simulations of the timing of cloud affect the simulation of top-of-atmosphere energy balance.

Now, the most comprehensive tools available to predict future global climate change are Earth system models (ESMs), which simulate the physics of Earth’s atmosphere, oceans, land surface, and ice processes. To allow predictions on a decadal scale, ESMs are run at a relatively low resolution (~1°), and thus, many smaller-scale physical processes that cannot be resolved are represented (or “parameterized”) by relatively simple equations meant to approximate the subgrid scale physics. Clouds are mediated by physics that occur across a wide range of scales, spanning global-scale dynamics (*5*–*9*) and micrometer-scale microphysics (*10*–*13*). Hence, many cloud-influencing processes must be parameterized within ESMs. Despite decades of advances in scientific understanding, clouds and their impacts on Earth’s radiation balance remain a leading source of uncertainty in our estimates of future climate change (*14*–*20*).

The SWCRE depends on the radiative properties of the clouds that are present and the amount of incident sunlight at a given time. Some types of clouds follow a diurnal cycle, where there is a preferential time of day that typically has maximum cloud cover and a time with minimum cloud cover. For example, stratocumulus clouds over subtropical and tropical oceans peak in the early morning. When the sun rises, the incident sunlight warms the cloud tops and increases the stability of the boundary layer. This reduces vertical transport of moisture from the surface, resulting in a decrease in cloud cover later in the day (*21*). There is also a diurnal cycle to convective clouds, which peak in the early morning over ocean and the late afternoon over land as a result of the impact of diabatic solar heating on planetary boundary layer buoyancy, surface convergence, and vertical mass flux (*22*–*25*). The timing of cloud occurrence is an important factor in determining the SWCRE because clouds occurring earlier or later in the day when solar angles are low will reflect less incident sunlight than those occurring near noon local time. However, as solar zenith approaches the horizon, there is an offsetting effect whereby cloud reflectance increases because of the photon path length required for sunlight to travel through a cloud (*26*).

Webb *et al.* (*27*) showed that across ESMs, there is substantial intermodel spread in the diurnal cycle of atmospheric liquid water path (LWP). This spread in the diurnal cycle may be inhibiting the accuracy of models’ SWCRE in simulations of the present-day and future climates. Webb *et al.* (*27*) also find that across ESMs, the LWP diurnal cycle changes little in response to global warming, even when the daily-mean LWP does change. Therefore, the model-simulated future SWCRE is directly related to the present-day cloud diurnal cycle. This point motivates investigation into the degree to which errors in the simulated LWP diurnal cycle affect SWCRE, even if the daily-mean LWP is simulated with high fidelity.

Webb *et al.* (*27*) do not take changes in aerosol concentration into account when predicting future changes in the LWP diurnal cycle. Smalley *et al.* (*28*) showed that increasing aerosols result in decreasing daytime LWP from enhanced entrainment drying of the boundary layer and increased nighttime LWP from precipitation suppression. Hence, the effective radiative forcing of aerosol cloud interactions also depends on the cloud diurnal cycle.

We examine the effect of errors in the simulated LWP diurnal cycle on SWCRE by comparing the observed diurnal cycle of cloud LWP from the Multisensor Advanced Climatology of Liquid Water Path [MAC-LWP; details of which are available in the study of Elsaesser *et al.* (*29*)] with output from ESMs. We institute an analytical model to quantify how much ESM bias in the LWP diurnal cycle contributes to that ESM’s SWCRE bias. However, because most models do not save sufficient output to calculate the LWP diurnal cycle, we also derive a method for quantifying the maximum possible effect of the cloud diurnal cycle timing (or phase shift) on the SWCRE. We then compare how the regions with a greater maximum possible effect of cloud diurnal cycle phase on SWCRE coincide with regions of high model SWCRE bias. We use output from ESMs that contributed to the sixth phase of the Coupled Model Intercomparison Project (CMIP6) (*30*), as well as an ensemble from version 3 of the Energy Exascale Earth System Model (E3SMv3) model (*31*) where model parameters were perturbed to test the range of possible climates simulated by the E3SMv3 model depending on parameter choice. Such ensembles are typically called perturbed parameter ensembles (PPEs), and more details on the E3SMv3 PPE are available in Materials and Methods. We use the E3SMv3 PPE to determine how much bias in SWCRE exists when the PPE is calibrated to replicate the observed daily-mean LWP.

## RESULTS

### Contribution of the LWP diurnal cycle to SWCRE bias

We develop a simple analytical model that approximates the SWCRE as proportional to the first harmonic of the grid-cell averaged LWP diurnal cycle multiplied by the solar cycle at a given location. This model is derived in detail in Materials and Methods. A benefit of this model is that it allows us to separate the SWCRE bias (${\text{SWCRE}}^{\prime }$) into its component parts: biases as a result of errors in the amplitude of the LWP diurnal cycle ($\text{SWCR}E_{A}^{\prime }$), the phase or timing of the LWP diurnal cycle ($\text{SWCR}E_{\text{phase}}^{\prime }$), the daily-mean LWP $\left(\text{SWCR}E_{\bar{\text{LWP}}}^{\prime }\right)$, the ways that the amplitude and phase biases combine nonlinearly ($\text{SWCR}E_{\text{NL}}^{\prime }$), and the residual (ϵ) from the approximation. The bias in SWCRE is calculated relative to observations from the Clouds and Earth’s Radiant Energy Systems Energy Balance and Filled (CERES-EBAF) dataset (*32*–*34*), and the first harmonic of the LWP diurnal cycle and mean LWP biases are relative to observational estimates from MAC-LWP

$$\begin{array}{c} \text{SWCR}E^{\prime }=\text{SWCR}E_{A}^{\prime }+\text{SWCR}E_{\text{phase}}^{\prime }\\ +\text{SWCR}E_{\bar{\text{LWP}}}^{\prime }+\text{SWCR}E_{\text{NL}}^{\prime }+\epsilon \end{array}$$ (1)

We recognize that cloud droplet size is also an important determinant of SWCRE (*35*) and is not captured in our simple model. In addition, in reality, cloud albedo (and, thus, SWCRE) is dependent on solar zenith angle. This effect has been estimated from observations and depends on three-dimensional cloud structures (*26*), which are parameterized crudely in ESMs. Hence, ESMs’ representation of the relation between cloud albedo and solar zenith angle may also contribute to SWCRE bias; however, examining such is beyond the scope of the present study. The interaction of SWCRE with both cloud droplet size and solar zenith angle represents potential biases in our simple model and contributes to the residual term captured in Eq. 1. We exclude such effects from this study to maintain the interpretability of Eq. 1. Also, the relation between SWCRE and the grid-cell averaged LWP is in reality a nonlinear function because of the decaying effect of increasing LWP as cloud opacity is approached, but in Materials and Methods, we show that SWCRE is well approximated as linear with the grid-cell averaged LWP.

The nonlinear term, on the other hand, should not be interpreted as an error in our simple model and does represent bias in the LWP diurnal cycle of the ESMs. The nonlinear term arises from the fact that the amplitude and phase terms do not combine linearly such that when biases in the phase and amplitude occur simultaneously, their combined influence on SWCRE results in a greater bias than what would occur if the phase and amplitude biases occur separately.

Only two ESMs published sufficiently high temporal resolution output on the Earth System Grid Federation CMIP6 database to calculate the diurnal cycle of LWP. We apply the decomposition of Eq. 1 to the Max Planck Institute Earth System Model 1.2 for the High Resolution intercomparison project (MPI-ESM1-2-HR) (*36*), and Canadian Earth System Model version 5 (CanESM5) (*37*). MPI-ESM1-2-HR is run at a 0.93° horizontal resolution with 95 vertical levels (*36*). CanESM5 is run at a 2.8° horizontal resolution and 49 vertical levels (*34*). The simulations were run as a part of the Atmospheric Model Intercomparison Project (AMIP) experiment, where both models were forced with climatological atmospheric constituents and run with prescribed sea surface temperature and sea ice to match observations (*38*).

**Fig. 1.**
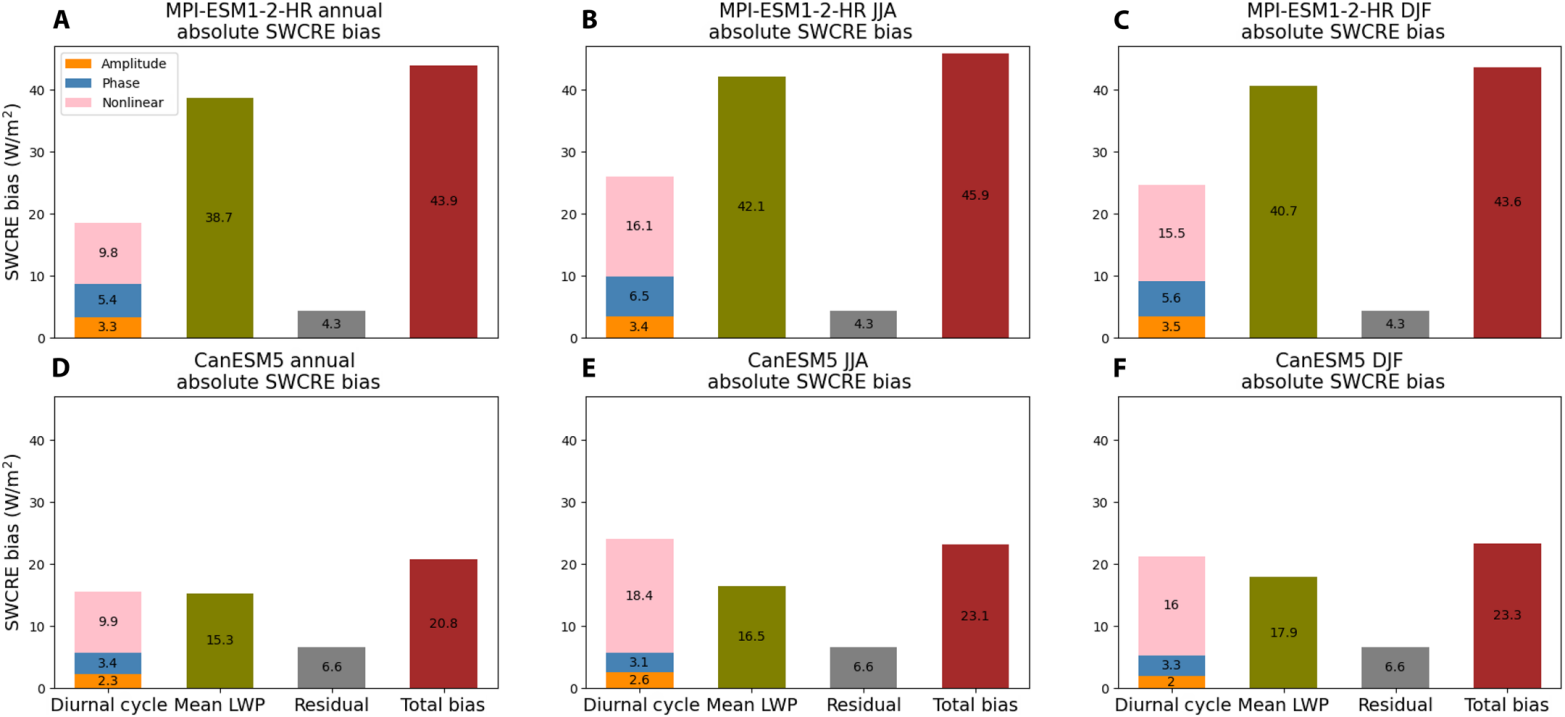
The absolute values of bias in SWCRE as a result of the LWP diurnal cycle and mean LWP are shown alongside the total SWCRE bias and residual of Eq. 1. They are shown for the MPI-ESM1-2-HR (**A** to **C**) and CanESM5 (**D** to **F**) output of the AMIP experiment. The annual mean is shown in [(A) and (D)], the JJA mean is shown in [(B) and (E)], and the DJF mean is shown in [(C) and (F)]. The numbers on each bar give the average bias for that term.

In Fig. 1, we show how each term in Eq. 1 contributes to the tropical and subtropical ocean mean total SWCRE bias in the annual; June, July, and August (JJA); and December, January, and February (DJF) means. We exclude LWP over land from this analysis because MAC-LWP does not estimate LWP over land. We limit our scope to regions between −40° and 40° latitude because, outside of this region, there is high incidence of mixed-phase clouds. While these clouds do contribute to the SWCRE, their ice fraction does not contribute to the LWP. This somewhat narrows the scope of our study and does not allow us to examine the diurnal cycle of polar clouds, which are typically mixed-phase or ice clouds. Future work could include a similar approach using ice water path, but current observations do not support global retrieval of the ice water path diurnal cycle. $\text{SWCR}E_{\text{NL}}^{\prime }$ is independent of the daily-mean LWP and only depends on the LWP diurnal cycle. As such, in Fig. 1, $\text{SWCR}E_{A}^{\prime }$, $\text{SWCR}E_{\text{phase}}^{\prime }$, and $\text{SWCR}E_{\text{NL}}^{\prime }$ are stacked to represent the total effect that biases in the LWP diurnal cycle have on the SWCRE. We note that the model residual is consistently smaller than the diurnal cycle and mean LWP contributions to SWCRE bias, suggesting that the effect of mechanisms excluded from our simple model (such as cloud droplet size) is comparatively small.

In both models, there is some similarity in each term of Eq. 1, as the LWP diurnal cycle effect on SWCRE bias is a substantial portion of the total SWCRE bias. This can be seen in Fig. 1 (A to C); in MPI-ESM1-2-HR, the mean LWP is the greatest source of SWCRE bias in the boreal winter (DJF), boreal summer (JJA), and annual means. The diurnal cycle effect is still relatively large, contributing 18.5, 26.0, and 24.6 W/m^2^ in the annual, JJA, and DJF means, respectively. Meanwhile, in CanESM5 (Fig. 1, D to F), the combined biases from the three diurnal cycle terms are the greatest source of SWCRE bias. This demonstrates that (i) these two models’ errors in the diurnal cycle should not be neglected if one hopes to improve the representation of SWCRE and (ii) the source of SWCRE bias is inconsistent across models. Maps showing the spatial patterns of each term in Eq. 1 and the combined effect of the diurnal cycle terms are available in figs. S1 to S3.

We note that the sum of the absolute value of the SWCRE bias because of each component of Eq. 1 is considerably more than the total SWCRE absolute bias (dark red bars of Fig. 1). This suggests compensating errors in the mean LWP and LWP diurnal cycle terms. To further interrogate this point, in figs. S4 to S6, we show maps of the actual value (not absolute value) SWCRE bias from each term in Eq. 1 and the total SWCRE bias. Such maps illustrate that there are considerable compensating errors across terms of Eq. 1 and cloud regimes such that the total SWCRE bias is less than the sum of the biases shown in Fig. 1.

### Phase susceptibility of the LWP diurnal cycle on SWCRE

To incorporate more models into our analysis of the LWP diurnal cycle’s contribution to SWCRE bias, we derive a metric relating the amplitude of the LWP diurnal cycle to the possible effect of the phase on the daily-mean SWCRE, hereafter referred to as phase susceptibility (in this case, phase refers to the diurnal cycle phase and does not relate to the cloud microphysical phase). This metric quantifies the potential impact of bias in the cloud diurnal phase on SWCRE but does not explicitly say anything about model bias. In addition, this metric only shows the potential effect of phase biases on SWCRE and does not show the potential for variations in amplitude. An important point about this metric is that it can be easily derived from observational estimates of the LWP diurnal cycle first harmonic, which were estimated from observations as part of the MAC-LWP dataset. MAC-LWP combines numerous spaceborne microwave radiometer retrievals of LWP into a global dataset. In doing so, it must correct for how the time of day that each sensor measures LWP affects monthly average estimates. MAC-LWP models the diurnal cycle of LWP as a second-order Fourier series (*29*). We use the first component of such to calculate the phase susceptibility

$$\text{Phase susceptibility}\left(\tilde{A},\phi_{\text{obs}}\right)\equiv \frac{\text{max}\left[\bar{\text{SWCRE}\left(t,\phi ,\tilde{A}\right)}\right]-\text{min}\left[\bar{\text{SWCRE}\left(t,\phi ,\tilde{A}\right)}\right]}{\bar{\text{SWCRE}\left(t,\phi_{\text{obs}},\tilde{A}\right)}}$$ (2)

where $\tilde{A}$ is the normalized amplitude of the cloud LWP diurnal cycle, ϕ is the phase of the LWP diurnal cycle, t is the time, and overbars denote temporal means. Details of this mathematical framework are available in Materials and Methods. Ultimately, the phase susceptibility represents the largest possible effect the diurnal cycle phase shift could have on SWCRE for the observed LWP mean and diurnal amplitude. The phase susceptibility is calculated entirely from observations, so it does not rely on any model data; however, it can be compared with model results to infer the potential importance of diurnal cycle errors on SWCRE bias. One important detail of this framework is that when calculating the phase susceptibility, we normalize by the SWCRE that corresponds to the observed LWP phase. We do so to ensure that regions of high phase susceptibility are not merely regions of high SWCRE (although, at times, this is the case). This means that the relationship between normalized diurnal cycle amplitude and phase susceptibility varies somewhat by location and the observed LWP phase ($\phi_{\text{obs}}$).

**Fig. 2.**
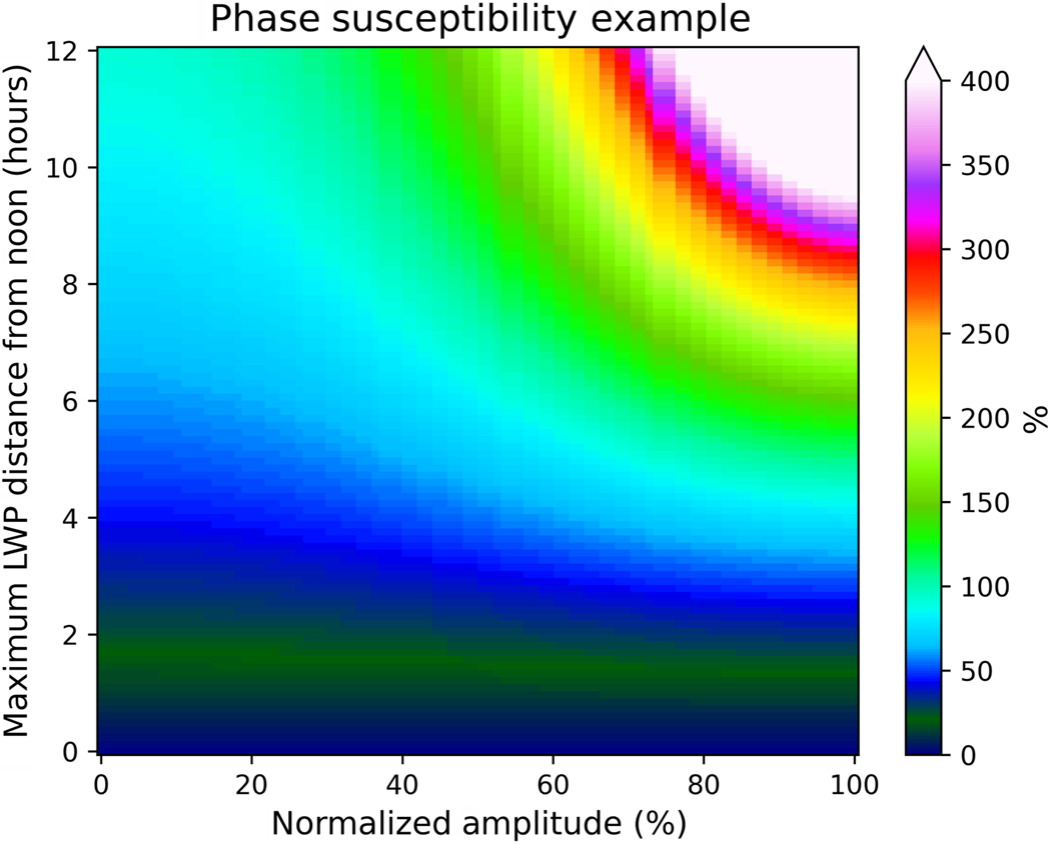
Examples of phase susceptibility (shaded) calculated from the LWP diurnal cycle represented by sinusoids that vary in amplitude from 0 to 100% of the mean and peak anywhere from 0 to 12 hours from noon.

In Fig. 2, we show the relationship between the LWP diurnal cycle normalized amplitude, phase, and phase susceptibility. We use a range of normalized amplitudes from 0 to 100% of the mean LWP and a range of phase shifts of maximum LWP at 0 to 12 hours away from noon (termed “distance” from noon). This results in a wide range of phase susceptibilities corresponding to each phase and amplitude combination. The instances with a stronger cloud diurnal cycle (greater amplitude) correspond with greater phase susceptibility. The relationship between amplitude and phase susceptibility becomes stronger with LWP, which maximizes further from noon. In addition, there are greater phase susceptibilities associated with nighttime LWP maxima (larger distance from noon). This occurs because nighttime LWP maxima correspond to lesser mean SWCRE such that shifting the diurnal cycle phase can have a greater fractional effect on the SWCRE than in instances where SWCRE peaks during the daytime.

**Fig. 3.**
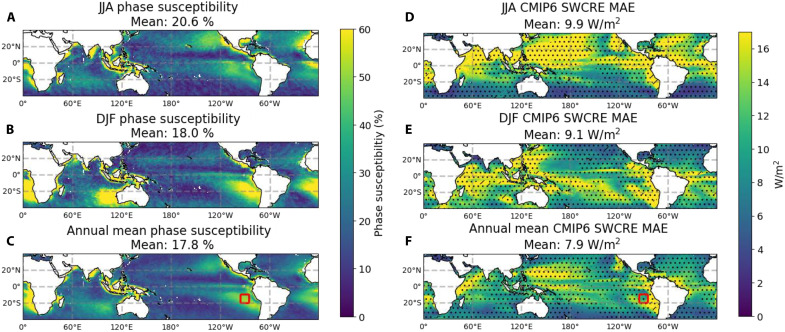
Maps of phase susceptibility alongside maps of SWCRE bias in CMIP6 models. (**A** to **C**) Maps of the phase susceptibility calculated from the MAC-LWP first harmonic of the diurnal cycle in JJA, DJF, and annual means, respectively. (**D** to **F**) Maps of the MAE of CMIP6 models’ SWCRE with respect to the CERES-EBAF SWCRE observations. Dotted stippling and hatching indicate regions where the multimodel mean MAE exceeds one or two standard deviations of the intermodel spread, respectively. The red squares in [(C) and (F)] show the area bounded by −20° to −10° latitude and 265° to 275° longitude.

This same framework is applied on a gridbox-by-gridbox basis to satellite-based estimates of the LWP diurnal cycle from the MAC-LWP dataset (*29*), and the results are shown in Fig. 3 (A to C). The greatest phase susceptibility occurs over ocean just west of Peru and northern Chile, Namibia, California, and Australia, with each region having greater phase susceptibility during their respective hemispheric winters. It is expected that these regions have high phase susceptibility because stratocumulus clouds occur frequently in all of these regions (*39*, *40*) and are known to have a strong diurnal cycle (*21*). Alongside the phase susceptibility in Fig. 3 (D to F), we show the mean absolute error (MAE) of SWCRE from 22 CMIP6 ESMs (model list available in table S1) relative to satellite observations from the CERES-EBAF dataset (*32*–*34*). Specifically, Fig. 3 (D to F) uses the SWCRE from AMIP model simulations (same model experiment as used in Fig. 1) (*38*).

We show the SWCRE MAE to point out that some regions with large CMIP6 model biases in SWCRE correspond to regions of high phase susceptibility. In particular, there is high SWCRE bias in the subtropics off the western coast of continents, and there is generally high SWCRE bias throughout the summer hemisphere, coinciding with locations of higher phase susceptibility in Fig. 3 (A to C). For example, in the annual mean, there is high phase susceptibility in the stratocumulus decks to the west of Peru. In the region bounded by −20° to −10° latitude and 265° to 275° longitude (marked with a red square in Fig. 3C), the phase susceptibility is 49.6% and the multimodel mean SWCRE MAE is 12 W/m^2^, which is about 20% of the observed SWCRE from CERES-EBAF of −59 W/m^2^. We show in fig. S7 that there is a statistically significant positive correlation between the spatial distribution of phase susceptibility and SWCRE MAE in the CMIP6 multimodel mean and provide discussion of this point in text S1. The correspondence between the SWCRE bias and phase susceptibility suggests that model bias in the timing of the LWP diurnal cycle may be one of the contributing mechanisms to the SWCRE bias. This hypothesis is especially pertinent over this multimodel ensemble, considering the substantial impact that the LWP diurnal cycle has on SWCRE in the two models examined in Fig. 1. We note however that this correspondence is far from perfect. There are many regions where phase susceptibility is low and SWCRE bias is high. SWCRE bias could also be due to biases in cloud droplet size or cloud ice. Subdaily model output is required to confirm the influence of the LWP diurnal cycle on SWCRE bias.

### SWCRE model bias when calibrating for the daily-mean LWP

In two ESMs, the diurnal cycle contributes SWCRE bias on the same order of magnitude as the mean LWP, and across a large ensemble of ESMs, some regions with large SWCRE bias correspond with large phase susceptibility. A natural question is whether this is due to biases in the mean amount of liquid cloud, which varies markedly across CMIP models (*41*), rather than the diurnal cycle. Considering that we do not have the output required to apply the Eq. 1 decomposition, we test the relative importance of the mean LWP amount by calibrating a PPE to simulate SWCRE bias in a model that correctly simulates the grid-cell mean LWP. This highlights SWCRE bias as a result of other mechanisms including the LWP diurnal cycle. We stress that it would be preferable to also calibrate the PPE to have the correct LWP diurnal cycle. However, the subdaily output of LWP is not available from a PPE because of data storage limitations.

**Fig. 4.**
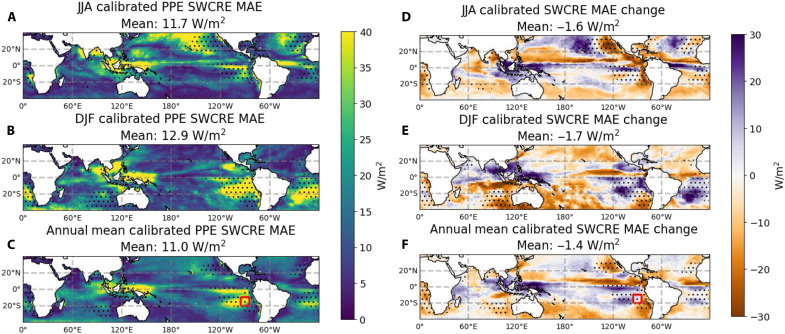
Maps of the SWCRE bias in the calibrated PPE and how SWCRE bias changes by calibrating the PPE to only include members with the most accurate mean LWP. (**A** to **C**) Maps of the SWCRE MAE compared with CERES-EBAF from the average of the 10 PPE members with the smallest RMSE of mean LWP. (**D** to **F**) Maps of the difference in SWCRE MAE compared with CERES-EBAF between the full-PPE mean and the average of the PPE members with the smallest RMSE of mean LWP. Stippling indicates regions where the phase susceptibility exceeds 30%. As in Fig. 3, the red squares in [(C) and (F)] show the area bounded by −20° to −10° latitude and 265° to 275° longitude.

We calculate the SWCRE bias from members of a PPE that have grid-cell mean LWP values close to the MAC-LWP observations. We use the PPE performed with E3SMv3 (*42*) where 25 atmospheric parameters were perturbed via Latin hypercube sampling with 237 ensemble members (*31*). We calibrate the PPE against the daily-mean LWP by selecting the 10 ensemble members with the smallest root mean square error (RMSE) of the model output total cloud LWP (including stratiform and convective cloud) compared with the MAC-LWP observational dataset. The RMSE is calculated from maps of LWP bias such that regional variations are taken into account as opposed to only the global mean LWP. The calibration of the PPE is detailed in Materials and Methods. We note that the cloud LWP estimated by MAC-LWP is estimated on the basis of the total LWP (rain and cloud LWP), and partitions between rain and cloud water are based on the sea surface temperature (*27*). This adds some caveat to our PPE calibration on the basis of the MAC-LWP dataset, as it remains to be tested how directly comparable MAC-LWP is with the model output LWP. However, considering that we are using this as an example of how models with low mean LWP bias can still exhibit SWCRE bias, this approximation is justifiable. If we were using MAC-LWP as an observational constraint or using it to tune a model, a more comprehensive use of a mask dictating which coincident MAC-LWP and model grid boxes can be compared in an analysis would be required. In Fig. 4, we show the calibrated-ensemble mean SWCRE MAE alongside the difference in SWCRE MAE between the calibrated-ensemble mean and the full PPE mean. Figure 4 (A to C) is meant to draw attention to the locations where high phase susceptibility (denoted by stippling) does or does not correspond with locations of high SWCRE bias in the subset ensemble. Meanwhile, Fig. 4 (D to F) shows the areas where there is reduced or increased SWCRE bias in the subset ensemble compared with the full PPE mean. The PPE reveals a stronger positive correlation between the SWCRE MAE and the phase susceptibility than the CMIP6 ensemble (see fig. S7 and text S1).

In all three time periods (JJA, DJF, and annual means), the calibrated PPE results in a smaller mean SWCRE bias than the uncalibrated PPE. Regions with phase susceptibility greater than 30% are stippled in all panels of Fig. 4 to highlight the overlap between biases and phase susceptibility. There is some correspondence between regions with high phase susceptibility and SWCRE bias in the calibrated ensemble. For example, in JJA, there is large bias in the Northern Pacific Ocean and west of California, and in DJF, there is large bias in the Subtropical Pacific and Atlantic Oceans to the west of Africa and South America. In the same region to the west of Peru as mentioned in the previous section (−20° to −10° latitude and 265° to 275° longitude), we find that there is an SWCRE MAE in the calibrated PPE of 38 W/m^2^, which is greater than the SWCRE MAE in the uncalibrated, full PPE (35 W/m^2^). In this region of high phase susceptibility, calibrating a model on the basis of the mean LWP therefore does not improve SWCRE bias. Hence, this SWCRE bias must be due to some other mechanism, which could be the LWP diurnal cycle or could also be the cloud droplet size distribution or three-dimensional scattering effects and bidirectional reflectance, which do not contribute to the phase susceptibility.

There are other regions with large SWCRE bias where the phase susceptibility is low, such as in the tropical western Pacific and along the Intertropical Convergence Zone that runs roughly along the equator and is a region of persistent surface convergence with frequent mesoscale convective systems (*43*, *44*). This may be because many of the deep convective clouds in the Intertropical Convergence Zone are ice or mixed-phase clouds and thus affect the SWCRE but do not contribute to the phase susceptibility calculated from MAC-LWP. This highlights the need for future work on developing diurnally resolved ice cloud observations, such as using the upcoming PolSIR (Polarized Submillimeter Ice-cloud Radiometer), as well as modeling studies on the diurnal cycle of deep convective clouds.

## DISCUSSION

### Summary and key points

Here, we use a method to separate the SWCRE bias of two ESMs into components: a bias arising from the LWP diurnal cycle and a bias arising from the error in their simulation of the mean LWP. Inaccurate simulations of LWP diurnal cycles in MPI-ESM2-1 contribute SWCRE biases that are at least 45% as large as the SWCRE biases arising from the error in the mean LWP. In CanESM5, the diurnal cycle of LWP accounts for greater bias in SWCRE than the mean LWP contribution. We find that biases in both the amplitude and phase of the LWP diurnal cycle cause notable contributions to the SWCRE bias, but the greatest contributor to SWCRE bias is the nonlinear combination of the phase and amplitude. This highlights the point that if modeling groups hope to improve the SWCRE biases of their ESMs, it is important to improve both the amplitude and phase of the LWP diurnal cycle. Despite these large errors in the LWP diurnal cycle, the global mean SWCRE MAE is far smaller than would be suggested by summing the errors as a result of the diurnal cycle with the errors resulting from daily-mean cloud. This is evidence that there are compensating errors within the ESMs, where errors at different locations have a canceling effect in the global mean, and there are errors in the daily-mean clouds that compensate errors in the diurnal cycle.

We additionally use a combination of observations and model output to identify, across a larger ensemble of ESMs, whether the timing of the cloud diurnal cycle is a possible source of error in model-simulated present-day SWCRE. We find that the regions where the SWCRE is most susceptible to variations in the phase of the LWP diurnal cycle coincide with regions of high SWCRE bias across CMIP6 models. The results from the high-temporal resolution output from MPI-ESM1-2-HR and CanESM5 provide an example of how the LWP diurnal cycle is an important contributor to SWCRE bias in these two ESMs. In a larger ensemble of ESMs (which did not save necessary output for the explicit decomposition), there are some regions where there is high phase susceptibility from MAC-LWP observations and also high SWCRE bias across CMIP6 models, and there is a statistically significant positive correlation between SWCRE bias and phase susceptibility. This indicates that more models than MPI-ESM1-2-HR and CanESM5 may suffer from SWCRE bias because of deficiencies in their LWP diurnal cycles; however, further research using simulations that save subdaily output is required to confirm this hypothesis. Last, by calibrating the E3SMv3 PPE to only include the ensemble members with the smallest bias in the mean LWP, we still find ssubstantial bias in SWCRE. Similar to the CMIP6 ensemble, the calibrated PPE may also suffer from SWCRE bias because of the LWP diurnal cycle.

Some of the largest SWCRE biases occur in regions where stratocumulus clouds are common, which is also where phase susceptibility is the greatest. This highlights the importance of correctly simulating the timing of stratocumulus clouds. Many processes affect stratocumuli and when they might occur such as the entrainment drying of the boundary layer, radiative cooling rate at the cloud top, aerosol and cloud droplet nucleation, and large-scale dynamics such as subsidence rate and inversion strength (*5*, *45*, *46*). Investigating such mechanisms to improve ESMs’ simulation of stratocumulus cloud amount and thickness is already a popular area of study (*13*, *39*, *47*–*51*), but our results indicate that improving the fidelity of our ESMs’ simulations of SWCRE requires advances in how these mechanisms affect the timing of stratocumulus clouds as well.

Stratocumuli have a strong diurnal cycle where the LW cooling at the cloud top continuously destabilizes the boundary layer throughout the nighttime, and as the sun rises in the sky, insolation heats the cloud tops, which has a stabilizing effect, causing a decrease in LWP during the day (*45*). There are numerous reasons why ESMs may be misrepresenting this cycle, including errors in their autoconversion rates that can either exacerbate or dampen the overnight moistening of the boundary layer and incorrect cloud top entrainment rates that can cause either too much or too-little boundary layer desiccation from the free-tropospheric mixing that occurs preferentially during the daytime. There is also the “too few too bright” problem that has plagued generations of ESMs, where they simulate too little stratocumulus cloud and too much open-cell cumulus cloud, but their cumuli are more reflective than observed, resulting in relatively small errors in SWCRE (*52*, *53*). Open cumuli have a notably different diurnal cycle from stratocumuli (*54*), so misrepresenting the stratocumulus-cumulus transition could cause bias in the LWP diurnal cycle even if the diurnal cycle of each cloud regime is well represented.

### Implications for future science

These findings on how the LWP diurnal cycle affects SWCRE open the door to possibilities in geoengineering to mitigate anthropogenic climate change. Specifically, marine cloud brightening is a currently proposed method where aerosols are introduced into weakly precipitating cloud regimes (typically stratocumulus) to increase the number of cloud condensation nuclei, thereby increasing the reflectivity and lifetime of the clouds (causing more sunlight to be reflected by the clouds) (*55*–*57*). A cloud-resolving modeling study has shown that the effectiveness of marine cloud brightening depends on when the aerosol injection occurs (*48*). Smalley *et al.* (*28*) showed that increasing aerosols in stratocumulus regimes can have a substantial impact on the diurnal cycle of LWP. Our results suggest further research into how biases in ESMs’ diurnal cycle of LWP may be influencing their ability to simulate the effectiveness of marine cloud brightening. In addition, we have shown that variations in the timing of LWP can have a large impact on SWCRE, and as such, further work should investigate whether controlling the timing of aerosol emissions into stratocumulus regimes could be used to affect the timing of the LWP diurnal cycle to counteract climate change.

We show how, in two ESMs, the LWP diurnal cycle contributes to the SWCRE bias. However, for the other ESMs considered, we must speculate—on the basis of the susceptibility of the SWCRE to the LWP diurnal cycle—that the LWP diurnal cycle contributes to their SWCRE bias. We hope that these results motivate newfound focus on improving the diurnal cycle of LWP in models by addressing what parameterizations or new model physics could alleviate the biases in the LWP diurnal cycle. This may include observational and process-modeling studies to further understand the physics that affects the diurnal cycle of LWP and cloud. It could include future geostationary satellite observations, which observe the same region at all times of day. In addition, we encourage future modeling efforts to store more subdaily model output to allow for more accurate determination of the bias in SWCRE resulting from the timing of the LWP occurrence in a larger ensemble of models. We recognize that saving subdaily output can become expensive; hence, we recommend exploration into methods to save the diurnal cycle without requiring a burdensome amount of data storage. Alternative ways to save the diurnal cycle include saving the output on a coarsened horizontal grid or saving model output in 25-hour increments such that, over the course of a month, the full diurnal cycle is captured. Saving the output of the LWP diurnal cycle is especially important as some modeling centers are beginning to use observational benchmarks in their tuning process (such as SWCRE), and subdaily output is necessary to ensure that low SWCRE bias is not achieved through compensating errors (i.e., ensuring that we get the right answer for the right reasons).

One could imagine that there could be compensating positive and negative SWCRE biases in the various terms of Eq. 1 that could result in a small total SWCRE bias. We stress that observational constraints of ESMs should focus on observable metrics that relate to processes that are tractably related to CRE and cloud feedback, as opposed to deriving constraints from observed present-day CRE alone (*58*–*60*). We see in Fig. 1 that this is at least partially the case for both CanESM5 and MPI-ESM1-2-HR, where the total SWCRE bias is considerably less than the sum of the diurnal cycle and mean LWP components. This indicates that there are compensating errors such that it is unlikely that the LWP and LWP diurnal cycle respond to global warming in a realistic manner. Because of data availability limitations, we cannot test this hypothesis with a larger ensemble of models. However, one could imagine models tuned to have the correct top-of-atmosphere fluxes having compensating errors in the timing and mean LWP (that could also be counteracted by various other mechanisms such as cloud particle size, cloud fraction, and clear-sky albedo) such that the underlying mechanisms would be wrong even when the top-of-atmosphere fluxes create the appearance of an accurate model. Because of these compensating errors, fixing the cloud diurnal cycle alone (and not changing any other model physics) would likely not solve the SWCRE bias. However, the biases in the LWP diurnal cycle shown in Fig. 1 are evidence that there are faults in the underlying physics hidden by compensating errors. Such that model improvement requires correcting multiple mechanisms to ensure that a model is getting the right answers for the right reasons, and our results show that the cloud diurnal cycle needs to be one of the mechanisms considered. There have been numerous studies on ESM bias in clouds and SWCRE and how they affect future climate (*58*–*65*). Our results suggest the need for similar studies on the LWP diurnal cycle to further understand its mediating physics and impact on simulations of future climate. Correcting the mean LWP bias is a necessary but insufficient step toward eliminating SWCRE bias; improving the representation of the LWP diurnal cycle must be considered as well.

## MATERIALS AND METHODS

### E3SM PPE

This work uses the Nephele PPE run in version 3 of the Energy Exascale Earth System Model (E3SMv3) (*42*). E3SMv3 was run in an atmosphere-only configuration for 24 months starting from 1 January 2010. The PPE simulations were run in preindustrial and present-day scenarios using aerosol forcings from 1850 or 2010, respectively; only the present-day simulations are used in this study. Free-tropospheric winds were nudged toward reanalysis with a 6-hour relaxation time. A total of 25 parameters were perturbed across the E3SMv3 microphysics, convective microphysics, and aerosol schemes to address parametric uncertainty related to aerosol-cloud interactions and adjustments. These parameters were perturbed simultaneously using Latin hypercube sampling (*66*) across 250 ensemble members. Fourteen ensemble members were excluded because of errors in the output, leaving an ensemble of 237 members including the default E3SMv3 tuning. More details on the Nephele E3SMv3 PPE are described by Nugent *et al.* (*31*).

### SWCRE bias decomposition

SWCRE is defined as the difference between the upwelling SW flux under all-sky $\left(F_{\text{all}}\right)$ and clear-sky ($F_{\text{clear}}$) conditions

$$\text{SWCRE}\left(t\right)=F_{\text{all}}-F_{\text{clear}}$$ (3)

$F_{\text{all}}$ can be expressed as a function of the upwelling SW flux under cloudy conditions ($F_{\text{cld}}$), $F_{\text{clear}}$, and the fractional occurrence of cloud (CF), as shown in Eq. 4

$$F_{\text{all}}=F_{\text{clear}}\left(1-\text{CF}\right)+F_{\text{cld}}\text{CF}$$ (4)

Inserting Eq. 4 into Eq. 3 yields a new expression for SWCRE.

$$\text{SWCRE}=\text{CF}\left(F_{\text{cld}}-F_{\text{clear}}\right)$$ (5)

Assuming plane-parallel conditions (an assumption typically made by ESM radiation schemes), the upward flux under cloudy conditions is equal to the product of the incident downwelling insolation flux ($S_{c}$) and the cloudy-sky albedo ($\alpha_{\text{cld}}$), as shown in Eq. 6

$$F_{\text{cld}}=S_{c}\alpha_{\text{cld}}$$ (6)

Then, the SWCRE can be expressed by Eq. 7, where $\alpha_{\text{clear}}$ is the clear-sky albedo; we find that SWCRE is a linear function of $S_{c}$, CF, and the difference in albedo between cloudy and clear-sky conditions

$$\text{SWCRE}=S_{c}\text{CF}\left(\alpha_{\text{cld}}-\alpha_{\text{clear}}\right)$$ (7)

Here, we are concerned with how biases in the diurnal cycle of clouds affect biases in SWCRE; hence, some assumptions are required to estimate how the diurnal cycle of clouds affects Eq. 7. Specifically, we know that CF and $\alpha_{\text{cld}}-\alpha_{\text{clear}}$ are related to the grid-cell averaged LWP, where the fraction of a grid cell containing liquid condensate equates to the liquid portion of CF, and the amount of liquid condensate contained in clouds relates to $\alpha_{\text{cld}}-\alpha_{\text{clear}}$. We approximate that these relationships can be well represented by a first-order Taylor series whereby the SWCRE is proportional to the LWP multiplied by the solar cycle at a given location (Eq. 8)

$$\text{SWCRE}≅C_{1}S_{c}\text{LWP}+\epsilon_{1}$$ (8)

where $\epsilon_{1}$ represents the residual of the linear model, and $C_{1}$ is the constant of proportionality between SWCRE and the LWP multiplied by the incident sunlight ($C_{1}=\frac{\bar{\text{SWCRE}}}{\bar{S_{c}\text{LWP}}}$, where overbars denote temporal averaging). $C_{1}$ is calculated from the mean SWCRE and LWP at each model grid cell. As such, the relation between SWCRE and LWP varies with location and is different for each model.

We emphasize that using grid-cell averaged LWP to approximate $\text{CF}\left(\alpha_{\text{cld}}-\alpha_{\text{clear}}\right)$ is advantageous over the cloud fraction because it incorporates information about the cloud fraction and cloud thickness into the model and does not rely upon any specific definition of cloud fraction, which can vary depending on the observing platform and ESM scheme (*67*).

Validation for the relationship in Eq. 8 is provided in fig. S8 where we show that the variations in LWP capture the variability of SWCRE very well when multiplied by the solar cycle. In Eq. 9, we approximate the LWP diurnal cycle as a first-order harmonic; A and ϕ are the amplitude and phase derived by fitting a first-order harmonic of a 24-hour period (ω=π/12 hours) to the LWP at each grid cell, respectively; and $\bar{\text{LWP}}$ is the time-averaged LWP. $\epsilon_{1}$ is the residual from the assumption that SWCRE is proportional to the product of LWP and the solar cycle. $\epsilon_{2}$ is the residual from the assumption that the LWP can be modeled accurately as a first-order harmonic

$$\text{LWP}≅A\text{cos}\left[\omega \left(t-\phi \right)\right]+\bar{\text{LWP}}+\epsilon_{2}$$ (9)

The full equation for the approximated SWCRE is as follows. ϵ is the residual from the approximated fit and includes the residual from both the approximations in Eqs. 8 and 9

$$\text{SWCR}E_{\text{approx}}=C_{1}S_{c}\left\{A\text{cos}\left[\omega \left(t-\phi \right)\right]+\bar{\text{LWP}}\right\}+\epsilon$$ (10)

We can then take the anomaly of SWCRE relative to observations from CERES-EBAF and write the linear effect on SWCRE bias from varying each term. Such decomposition is provided in the following equations where the notation ${\left[\cdot \right]}_{\text{obs}}$ indicates an observed variable and ${\left[\cdot \right]}_{\text{mod}}$ indicates model output

$$\text{SWCR}E^{\prime }=\text{SWCR}E_{A}^{\prime }+\text{SWCR}E_{\text{phase}}^{\prime }+\text{SWCR}E_{\bar{\text{LWP}}}^{\prime }+\text{SWCR}E_{\text{NL}}^{\prime }$$ (11)

$$\text{SWCR}E_{A}^{\prime }=\bar{C_{1}S_{c}\left(A_{\text{mod}}-A_{\text{obs}}\right)\text{cos}\left[\omega \left(t-\phi_{\text{obs}}\right)\right]}$$ (12)

$$\text{SWCR}E_{\text{phase}}^{\prime }=\bar{C_{1}S_{c}A_{\text{obs}}\left\{\text{cos}\left[\omega \left(t-\phi_{\text{mod}}\right)\right]-\text{cos}\left[\omega \left(t-\phi_{\text{obs}}\right)\right]\right\}}$$ (13)

$$\text{SWCR}E_{\bar{\text{LWP}}}^{\prime }=\bar{C_{1}S_{c}\left(\bar{\text{LW}P_{\text{mod}}}-\bar{\text{LW}P_{\text{obs}}}\right)}$$ (14)

As previously stated, $\text{SWCR}E_{\text{NL}}^{\prime }$ is the nonlinear combination of A, ϕ, and $\bar{\text{LWP}}$ and is calculated as the residual of the linearized decomposition, as follows

$$\begin{array}{c} \text{SWCR}E_{\text{NL}}^{\prime }=\bar{C_{1}S_{c}\left\{\bar{\text{LW}P_{\text{mod}}}-\bar{\text{LW}P_{\text{obs}}}+A_{\text{mod}}\text{cos}\left[\omega \left(t-\phi_{\text{mod}}\right)\right]-A_{\text{obs}}\text{cos}\left[\omega \left(t-\phi_{\text{obs}}\right)\right]\right\}}\\ -\left(\text{SWCR}E_{A}^{\prime }+\text{SWCR}E_{\text{phase}}^{\prime }+\text{SWCR}E_{\bar{\text{LWP}}}^{\prime }\right) \end{array}$$ (15)

We point out that the first two terms of Eq. 15 $\left[C_{1}S_{c}\left(\bar{\text{LW}P_{\text{mod}}}-\bar{\text{LW}P_{\text{obs}}}\right)\right]$ are equivalent to $\text{SWCR}E_{\bar{\text{LWP}}}^{\prime }$. As such, the nonlinear term is only a function of the amplitude and phase of the diurnal cycle and is independent of the mean LWP

$$\begin{array}{c} \text{SWCR}E_{\text{NL}}^{\prime }=\bar{C_{1}S_{c}\left\{A_{\text{mod}}\text{cos}\left[\omega \left(t-\phi_{\text{mod}}\right)\right]-A_{\text{obs}}\text{cos}\left[\omega \left(t-\phi_{\text{obs}}\right)\right]\right\}}\\ -\left(\text{SWCR}E_{A}^{\prime }+\text{SWCR}E_{\text{phase}}^{\prime }\right) \end{array}$$ (16)

In addition, in Fig. 1, we show the residual of the model (ϵ), which describes the inaccuracies of the two underlying assumptions in this model in Eqs. 8 and 9

$$\epsilon =\text{SWCR}E_{\text{mod}}-\text{SWCR}E_{\text{approx},\text{mod}}$$ (17)

These equations provide the mathematical framework for the SWCRE bias decomposition in Fig. 1.

### Phase susceptibility mathematical model

We quantify the maximum possible impact that the timing of the LWP diurnal cycle could have on the SWCRE through an adjustment to the previously established mathematical framework to approximate SWCRE. Beginning with Eq. 10, we then calculate the full possible range of SWCRE that occurs for all possible values of ϕ by using 1000 equally spaced values of ϕ between 0 and 24 and calculating the normalized LWP (denoted with tilde) as

$$\tilde{\text{LWP}\left(t,\phi \right)}\equiv \frac{\text{LWP}\left(t,\phi \right)}{\bar{\text{LWP}}}$$ (18)

such that

$$\tilde{\;\text{LWP}\left(t,\phi \right)}=\tilde{A}\text{cos}\left[\omega \left(t-\phi \right)\right]+1$$ (19)

where $\tilde{A}\equiv =\frac{A}{\bar{\text{LWP}}}$. Using this approximation, $\tilde{A}$ is available globally over oceans from the MAC-LWP satellite retrieval. We then calculate the range of SWCRE from variations in ϕ by calculating the LWP diurnal cycle and, thus, SWCRE from all possible values of ϕ.

Last, we define the phase susceptibility $\left[\text{PS}(\tilde{A},\phi_{\text{obs}})\right]$ from the SWCRE corresponding to each phase shift as the maximum possible range that the time-averaged SWCRE can have across all possible phase shifts

$$\text{PS}\left(\tilde{A},\phi_{\text{obs}}\right)\equiv \frac{\text{max}\left[\bar{\text{SWCRE}\left(t,\phi ,\tilde{A}\right)}\right]-\text{min}\left[\bar{\text{SWCRE}\left(t,\phi ,\tilde{A}\right)}\right]}{\bar{\text{SWCRE}\left(t,\phi_{\text{obs}},\tilde{A}\right)}}$$ (20)

We normalize the phase susceptibility by the observed SWCRE corresponding to the observed phase of the first harmonic of the cloud diurnal cycle, $\phi_{\text{obs}}$, such that the phase susceptibility is dimensionless and directly comparable to the normalized model error.

$\tilde{A}$ has some value between 0 and 1, so to derive the relationships shown in Fig. 2, we use 100 linearly spaced samples of $\tilde{A}$ to calculate the phase susceptibility. To calculate the phase susceptibility on the basis of observations, we approximate that the SWCRESc linearly correlates with LWP. Then, one must simply replace $\tilde{A}$ with the observed normalized amplitude of the LWP diurnal cycle, which is estimated globally over oceans from the MAC-LWP dataset (*29*); however as previously noted, we limit the scope of this study to the tropics and subtropics because of the high incidence of mixed phase and ice cloud in the extratropics.

### Perturbed parameter ensemble calibration based on the mean LWP

We use the PPE of E3SMv3 to select only the ensemble members that most accurately simulate mean LWP. This is done by selecting the 10 ensemble members with the lowest LWP RMSE compared with the observation from MAC-LWP. The RMSE is calculated following Eq. 21, where area is the area of a given model grid cell, $\text{LW}P_{\text{mod}}$ is the LWP in the ESM, and $\text{LW}P_{\text{obs}}$ is the observed LWP. The RMSE is calculated from grid cells over ocean between −40° and 40° latitude

$$\text{RMSE}=\sqrt{\frac{\sum_{\text{lat}}\sum_{\text{lon}}{\left[\text{LW}P_{\text{obs}}\left(\text{lat},\text{lon}\right)-\text{LW}P_{\text{mod}}\left(\text{lat},\text{lon}\right)\right]}^{2}*\text{area}}{\sum_{\text{lat}}\sum_{\text{lon}}\text{area}}}$$ (21)

This metric incorporates regional variations into the global error metric. As such, the 10 ensemble members chosen do not necessarily have the lowest global mean LWP bias but do have the lowest sum of all regional biases.

## Supplementary Materials

### This PDF file includes:

Text S1 Figs. S1 to S8 Table S1 References
