# Supplementary material for: Global models predict clouds at the wrong time of day: Does it matter for radiation and climate?
Source: Sci Adv. 2025 Dec 12;11(50):eady3236. doi: 10.1126/sciadv.ady3236 (PMC13142753; doi:10.1126/sciadv.ady3236)
Supplement: Supplementary file 1 — Text S1 Figs. S1 to S8 Table S1 References [file sciadv.ady3236_sm.pdf]

Science Advances
AAAS

Supplementary Materials for

Global models predict clouds at the wrong time of day: Does it matter for radiation and climate?

Travis Aerenson *et al.*

Corresponding author: Travis Aerenson, taerenso@uwyo.edu

Sci. Adv. **11**, eady3236 (2025)
DOI: 10.1126/sciadv.ady3236

This PDF file includes:

Text S1
Figs. S1 to S8
Table S1
References

Text S1. Regression of phase susceptibility on SWCRE MAE.

In the main text we show some visual correspondence between the phase susceptibility and SWCRE MAE in two datasets: the CMIP6 multi-model mean, and the E3SMv3 PPE ensemble mean. To expand on the correspondence shown schematically in the main text, we perform Spearman rank regression on the spatial distribution of the phase susceptibility and SWCRE MAE. In **Fig. S7** we show the Spearman rank correlation coefficients (hereafter SRCC) of each of the CMIP6 models and E3SMv3 PPE ensemble members. We find statistically significant to 95% confidence positive SRCCs for 185 out of 237 (79%) of the E3SMv3 PPE ensemble members, and 13 out of 21 (62%) CMIP6 models included in this study.

While this statistically significant correlation is supportive of our main points, we also emphasize that correlation between SWCRE MAE from models with the phase susceptibility from observations is a very high bar to indicate some contribution to the SWCRE MAE from the LWP diurnal cycle. There are many factors that can influence SWCRE that are independent of the LWP diurnal cycle, such that one would not expect perfect correspondence (SRCC of one) between SWCRE MAE and phase susceptibility. Not only does a strong correlation rely upon significant biases from the LWP diurnal cycle, but it also necessitates that the ESMs produce the correct atmospheric circulations such that each cloud regime is simulated in the correct locations relative to observations.

The E3SMv3 PPE was run with the free-tropospheric winds nudged to match reanalysis, so there are no biases in dynamics of the ensemble. As one might expect, this nudged ensemble has on average higher SRCCs than the CMIP6 ensemble (which was not run with atmospheric nudging). This difference between the PPE and CMIP6 ensemble highlights the effect that dynamical biases can have on the relation between phase susceptibility and SWCRE MAE.

We aim to understand the effect of ESM dynamical biases on the SRCCs in **Fig. S7**. In addition to using the phase susceptibility derived from MAC-LWP observations, we calculate the phase susceptibility from the model climatology for CanESM5 and MPI-ESM1-2-HR. We calculate the SRCC of the ESM SWCRE MAE with observationally derived phase susceptibility and with the phase susceptibility derived from the actual climatology within that ESM. This provides some quantification for how biases can dampen the relation between SWCRE and phase susceptibility for the other CMIP6 models. In a perfect world, we would do all the analyses using the phase susceptibility from the CMIP6 models, however, most did not save the sub-daily output that is needed to calculate phase susceptibility.

CanESM5 and MPI-ESM1-2-HR are among the CMIP6 models with the weakest SRCCs. We show in **Fig. 1** of the main text that there are significant biases in the LWP diurnal cycle of these two models, and it may be the case that there are larger biases in other CMIP6 models that lead to the stronger SRCC with phase susceptibility. In fact, the CMIP6 multi-model mean SWCRE MAE correlation with the observed phase susceptibility exceeds the SRCC found of CanESM5 and MPI-ESM1-2-HR with phase susceptibility from their own climates.

In MPI-ESM1-2-HR (dotted lines of **Fig. S7**), there is a very big difference between the SRCC found using the phase susceptibility calculated from observations and that calculated from the model's climate. There is very low correlation between the SWCRE MAE, and the phase susceptibility calculated from observations. However, there is much stronger correlation when the phase susceptibility from the model climate is used. Hence, it is possible for a model with a low rank correlation coefficient between SWCRE MAE and observed phase susceptibility to

have significant effects from the LWP diurnal cycle. 62% of the CMIP6 ESMs (and the multi-model mean) have statistically significant positive SRCCs, we deem it extremely unlikely that all such correlations are occurring without some contribution to the SWCRE MAE from biases in the LWP diurnal cycle. Thus, we conclude that bias in the LWP diurnal cycle is likely contributing to SWCRE bias across the CMIP6 models.

In summary, to achieve correlation between observationally derived phase susceptibility and SWCRE of a model, there is a hierarchy of conditions that must be met. Firstly, the model must simulate a climate state with cloud regimes that match observations reasonably well, such that the locations of high and low phase susceptibility in observations align with the corresponding cloud regime in the model. Secondly, those cloud regimes must be simulated in a sufficiently realistic way such that those with strong diurnal cycles in observations also have strong diurnal cycle in the model, and thirdly, there must be sufficient biases in the timing of the cloud diurnal cycle for the biases to have an impact on SWCRE despite other mechanisms that can contribute compensating errors. In our results, for most CMIP6 models and most members of the E3SMv3 PPE, we do find statistically significant SRCC between the SWCRE MAE and the phase susceptibility derived from observations. Additionally, we find that the ensemble with atmospheric nudging (which negates the first condition) typically has greater SRCC than the ensemble without nudging, and the CMIP6 ensemble mean SWCRE MAE has a greater SRCC with the phase susceptibility from observations than the SRCC from two example models' SWCRE MAE with phase susceptibility from their own climates (which negates the first and second conditions). Hence, we conclude that the third condition is sufficiently met such that despite the first and second conditions going unmet in the CMIP6 ensemble, there is a statistically significant correlation that is due to biases in the LWP diurnal cycle.

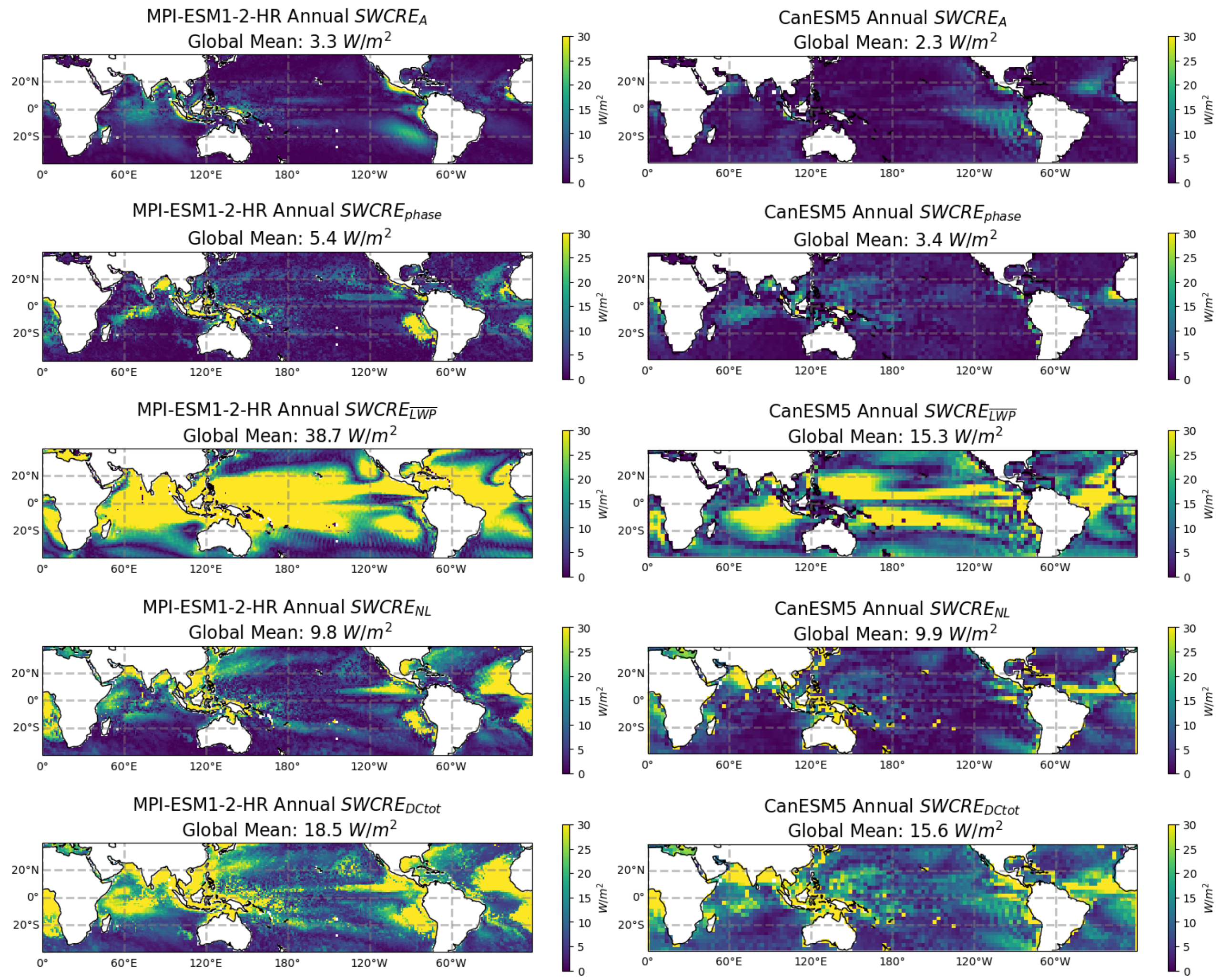

Figure S1.
Maps of the mean SWCRE absolute bias decomposed by each term in Equation 1, and the bottom panels show the sum of the amplitude, phase, and non-linear terms.

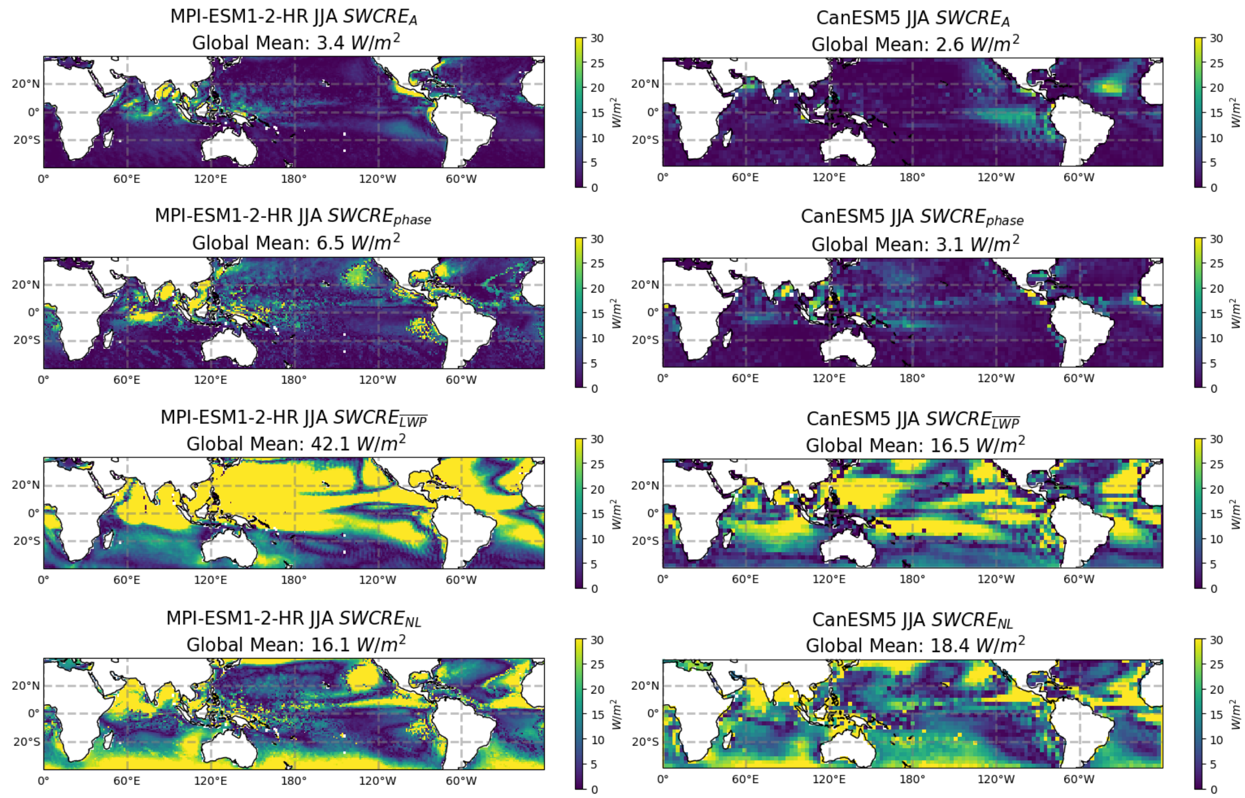

Figure S2.

Maps of the SWCRE absolute bias in JJA decomposed by each term in Equation 1, and the bottom panels show the sum of the amplitude, phase, and non-linear terms.

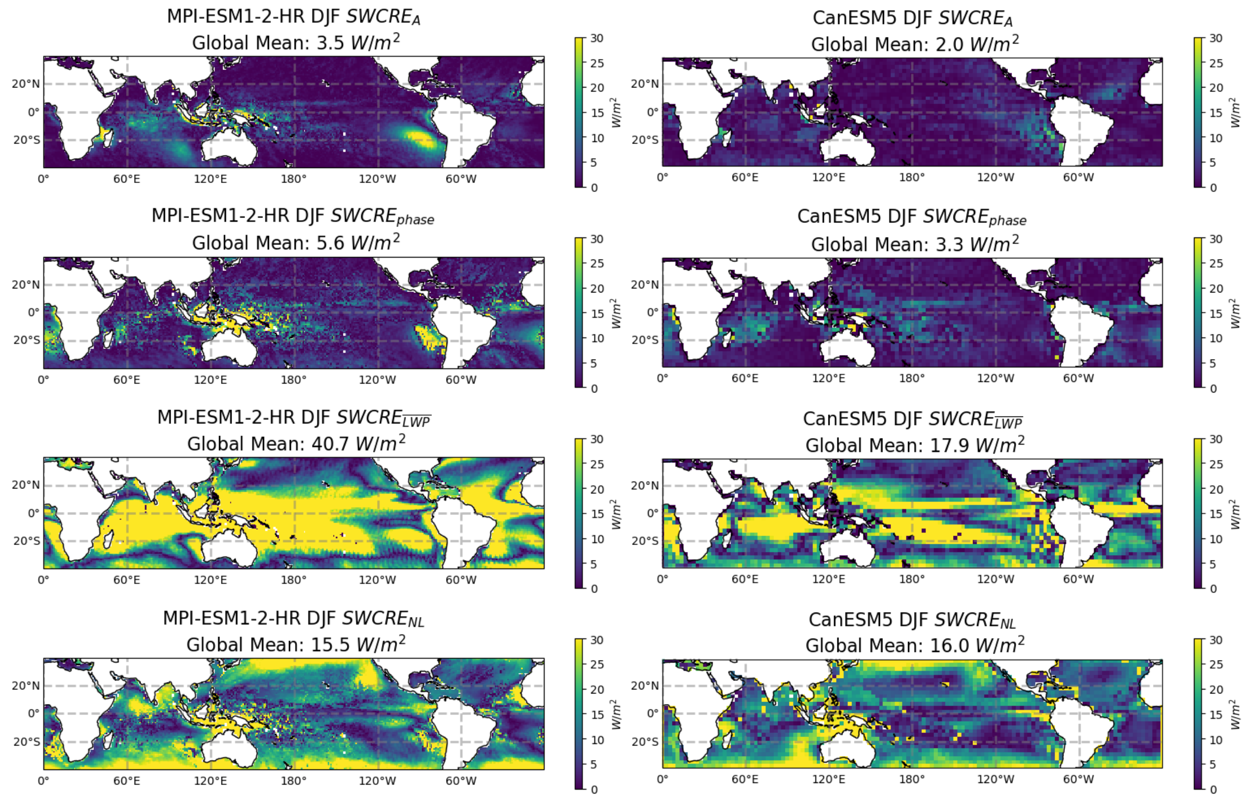

Figure S3.

Maps of the SWCRE absolute bias in DJF decomposed by each term in Equation 1, and the bottom panels show the sum of the amplitude, phase, and non-linear terms.

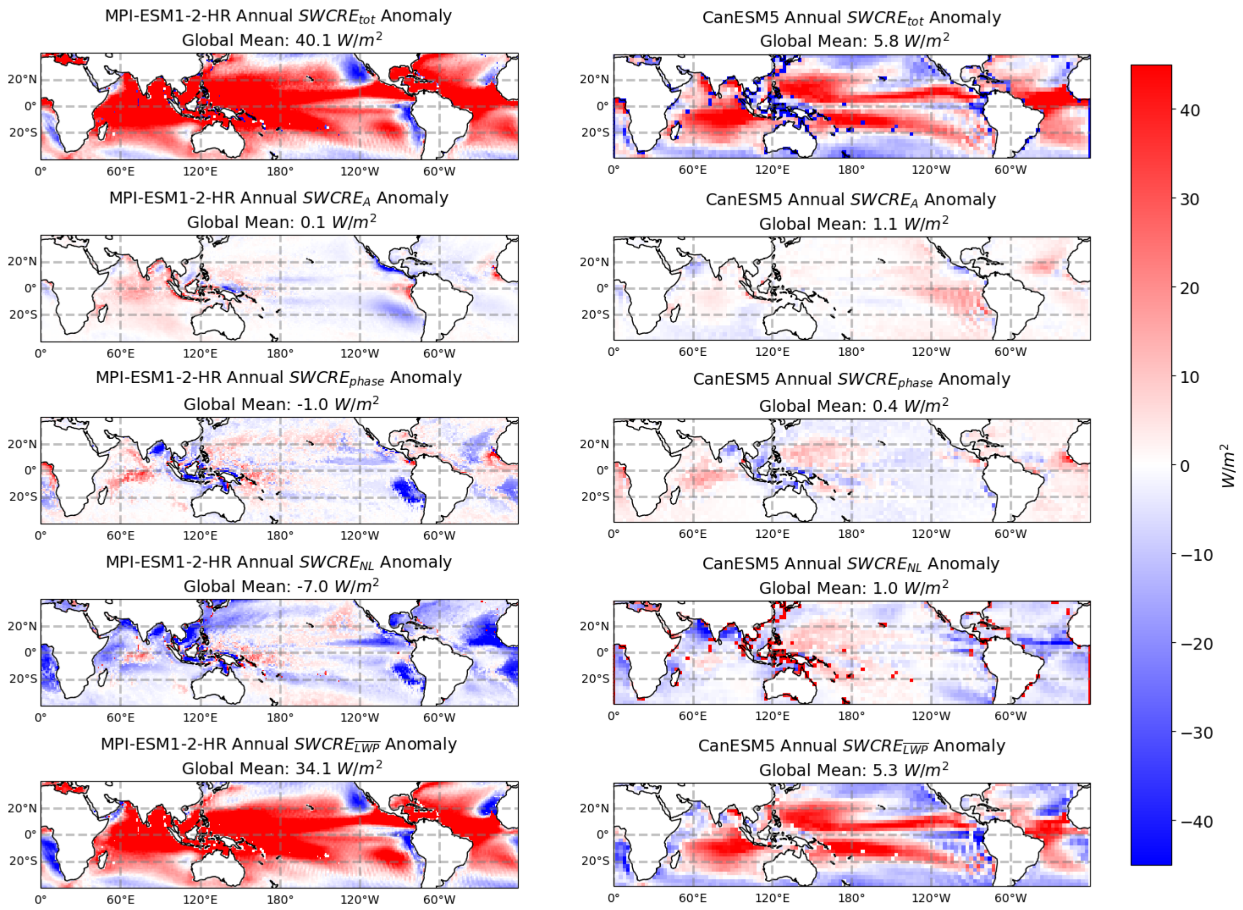

Figure S4.
Maps of the SWCRE annual mean anomaly due to each term in Equation 1 of the main text, as well as the total SWCRE anomaly for both CanESM5 and MPI-ESM2-HR.

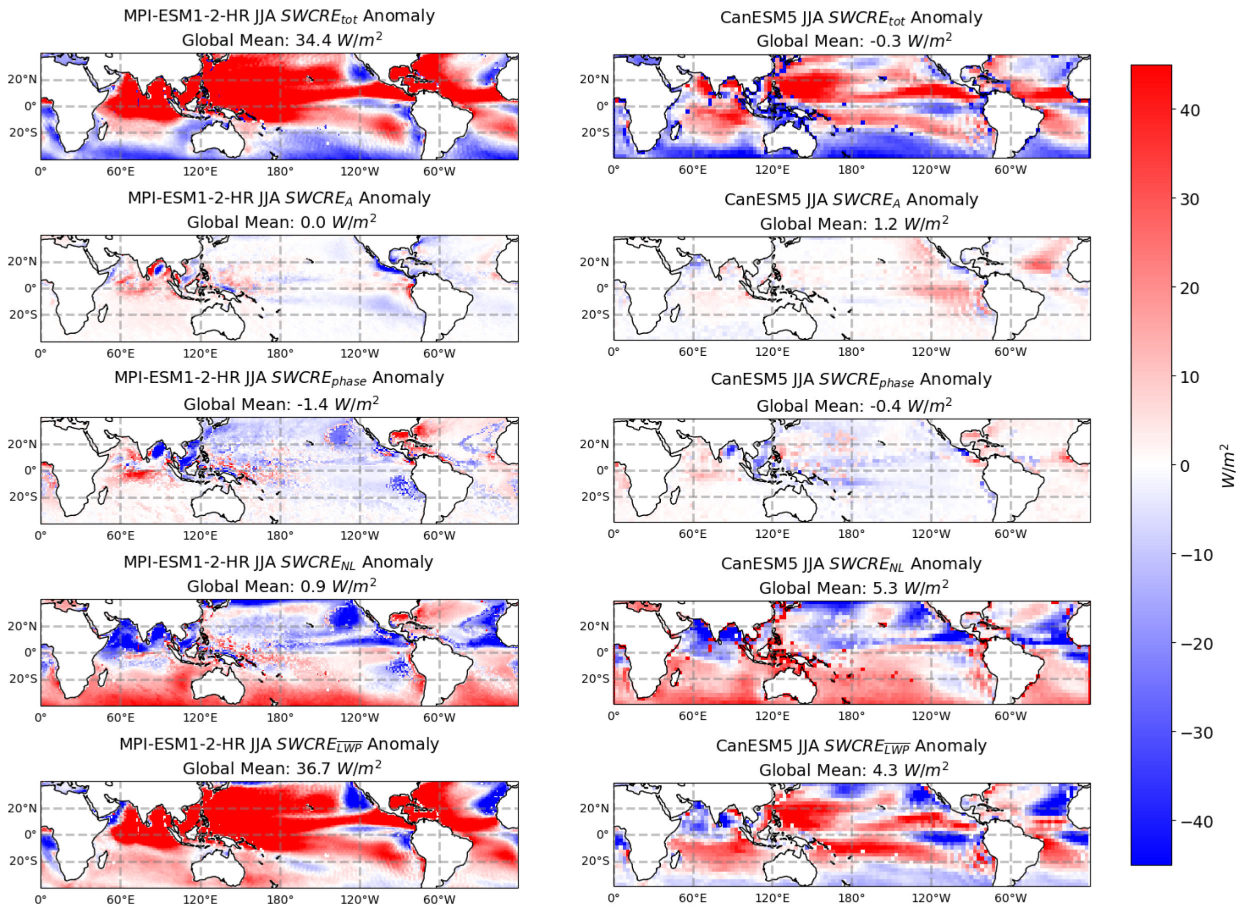

Figure S5.
Maps of the SWCRE JJA mean anomaly due to each term in Equation 1 of the main text, as well as the total SWCRE anomaly for both CanESM5 and MPI-ESM2-HR.

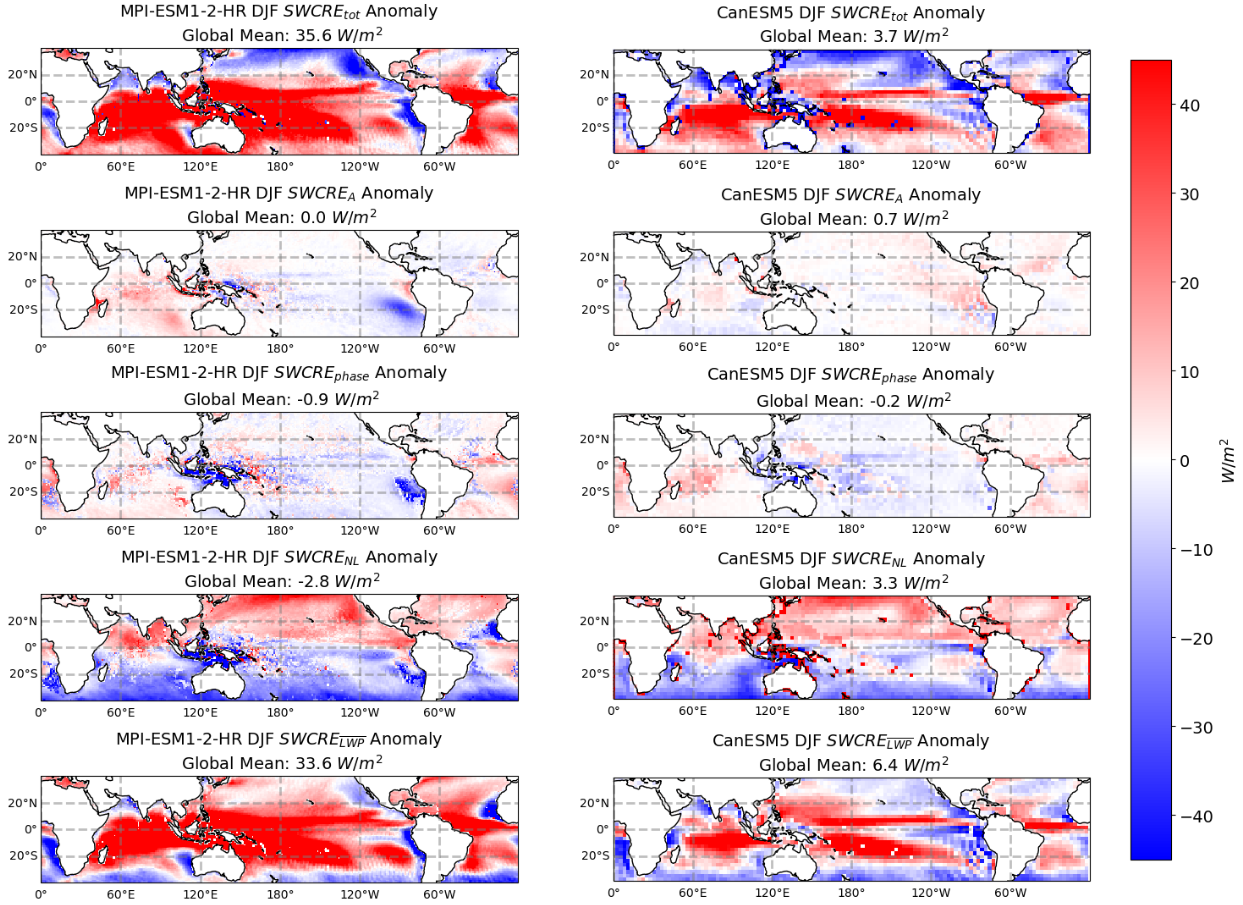

Figure S6.

Maps of the SWCRE DJF mean anomaly due to each term in Equation 1 of the main text, as well as the total SWCRE anomaly for both CanESM5 and MPI-ESM2-HR.

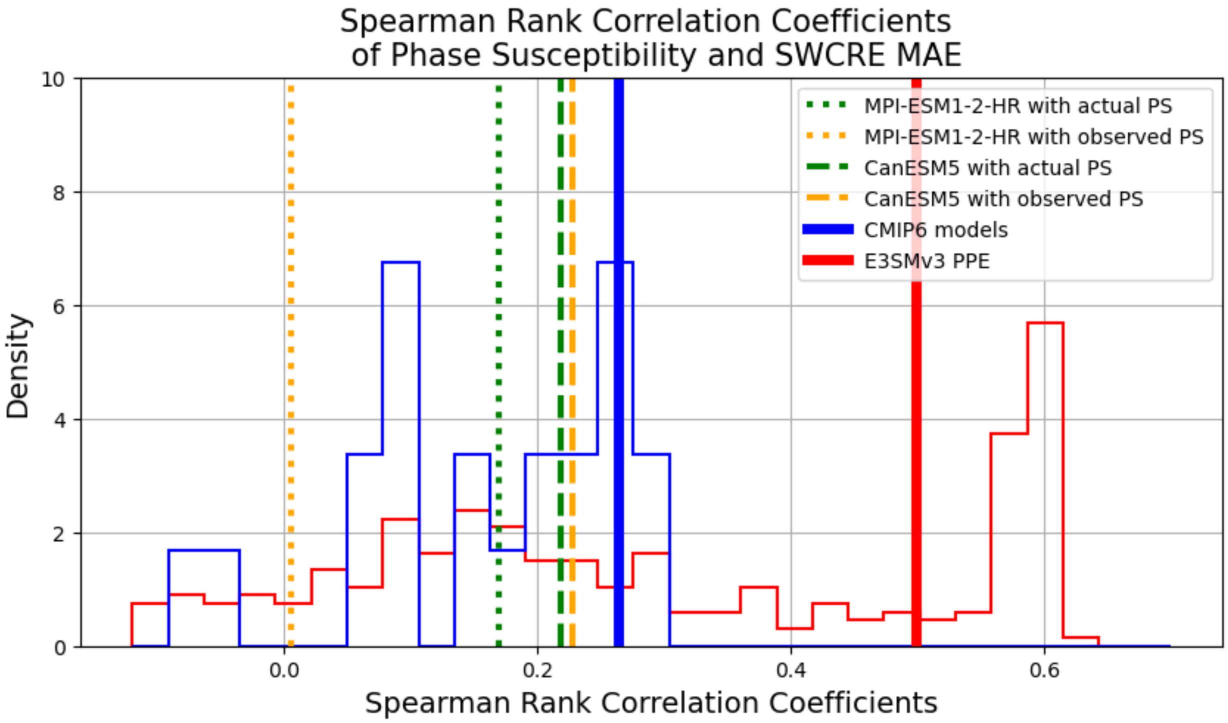

Figure S7.
Spearman rank correlation coefficients relating phase susceptibility with SWCRE MAE in each of the CMIP6 models (blue histogram), each member of the E3SMv3 PPE (red histogram), the CMIP6 multi-model mean (blue vertical line), the E3SMv3 PPE ensemble mean (red vertical line), and the two models for which we can calculate the phase susceptibility relative to their own climatology in orange and green lines for the regression with the observed phase susceptibility, and the model-simulated phase susceptibility respectively. All results were interpolated onto a 4° by 4° grid before performing the regression. Additionally, 100% of the models from both the CMIP6 and PPE ensembles with correlation coefficients greater than 0.1 yield statistically significant correlations to 95% confidence based on a one-tailed t-test. For the significance testing the effective number of independent samples is determined based on the spatial correlation using the framework of Bretherton et al. (*85*). The greater of the spatial correlations of the phase susceptibility and SWCRE MAE is used to calculate the effective number of independent samples for each regression.

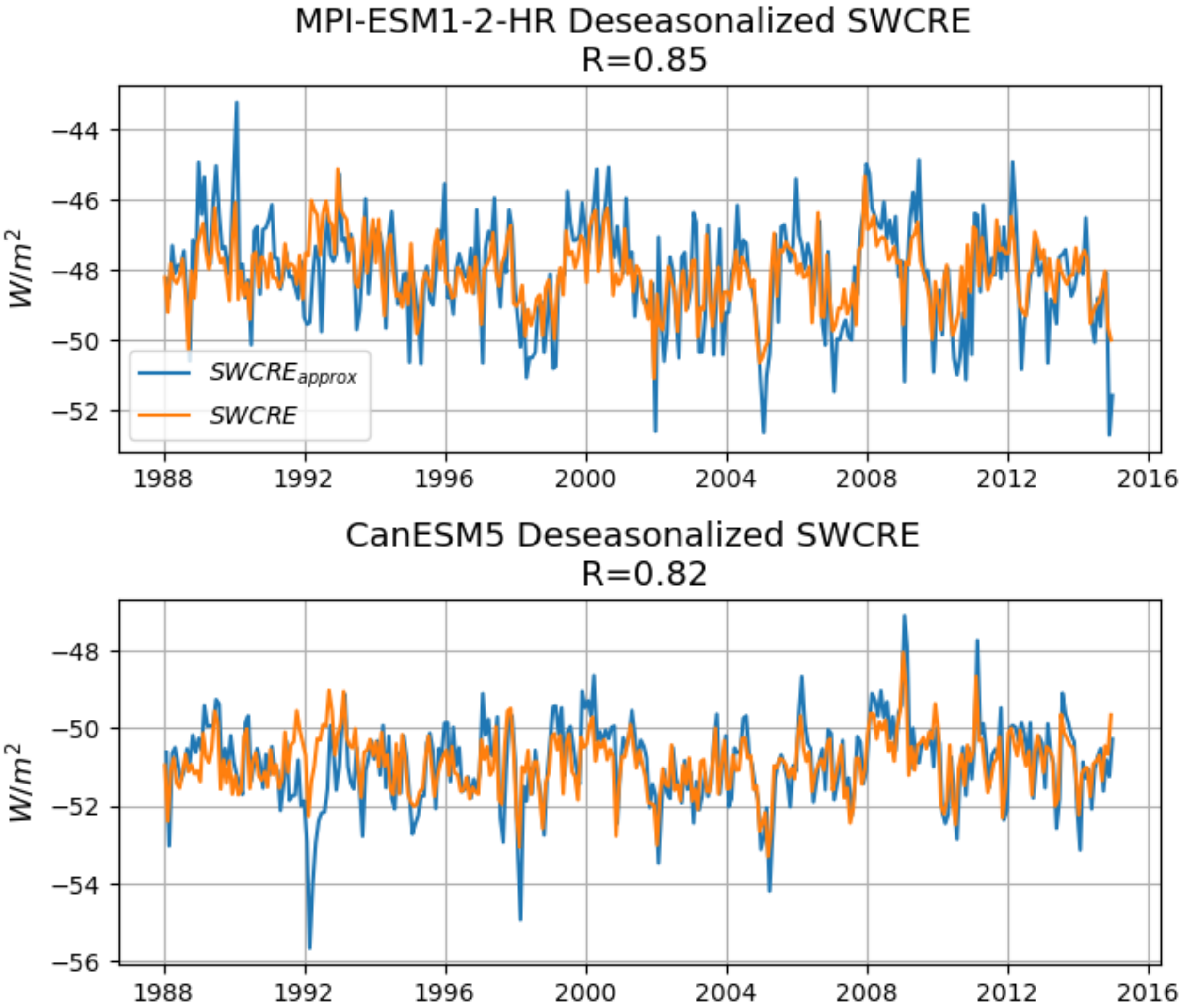

Figure S8.
Time series plots of the SWCRE from each model and the SWCRE approximated by Equation S1. In each plot the average seasonal cycle has been removed by subtracting the mean seasonal cycle of SWCRE. As with other plots, this shows the SWCRE over oceans bounded by -40° to 40° latitude.

Table S1. Full list of CMIP6 models used in **Fig. 3**, alongside their corresponding citation.

**Model**	**Citation**
BCC-CSM2-MR	Wu et al. (68)
BCC-ESM1	Wu et al. (69)
CAMS-CSM1-0	Rong et al. (70)
CESM2	Danabasoglu et al. (71)
CESM2-FV2	Danabasoglu et al. (71)
CESM2-WACCM	Danabasoglu et al. (71)
CESM2-WACCM-FV2	Danabasoglu et al. (71)
CNRM-CM6-1	Voldoire et al. (72)
CNRM-ESM2-1	Séférian et al. (73)
CanESM5	Swart et al. (37)
E3SM-1-0	Golaz et al. (74)
EC-Earth3-Veg	Döscher et al. (75)
GISS-E2-1-G	Kelley et al. (76)
HadGEM3-GC31-LL	Roberts et al. (77)
IPSL-CM6A-LR	Boucher et al. (78)
MIROC-ES2L	Hajima et al. (79)
MIROC6	Tatebe et al. (80)
MPI-ESM1-2-HR	Gutjahr et al. (36)
MRI-ESM2-0	Yukimoto et al. (81)
NESM3	Cao et al. (82)
SAM0-UNICON	Park et al. (83)
UKESM1-0-LL	Sellar et al. (84)

REFERENCES AND NOTES

1. S. C. Sherwood, S. Bony, O. Boucher, C. Bretherton, P. M. Forster, J. M. Gregory, B. Stevens, Adjustments in the forcing-feedback framework for understanding climate change. *Bull. Am. Meteorol. Soc.* **96**, 217–228 (2015).

2. R. A. Colman, B. J. McAvaney, On tropospheric adjustment to forcing and climate feedbacks. *Clim. Dyn.* **36**, 1649–1658 (2011).

3. J. M. Gregory, W. J. Ingram, M. A. Palmer, G. S. Jones, P. A. Stott, R. B. Thorpe, J. A. Lowe, T. C. Johns, K. D. Williams, A new method for diagnosing radiative forcing and climate sensitivity. *Geophys. Res. Lett.* **31**, L03205 (2004).

4. G. Myhre, E. J. Highwood, K. P. Shine, F. Stordal, New estimates of radiative forcing due to well mixed greenhouse gases. *Geophys. Res. Lett.* **25**, 2715–2718 (1998).

5. J. J. Van Der Dussen, S. R. De Roode, A. P. Siebesma, How large-scale subsidence affects stratocumulus transitions. *Atmos. Chem. Phys.* **16**, 691–701 (2016).

6. T. A. Myers, J. R. Norris, Observational evidence that enhanced subsidence reduces subtropical marine boundary layer cloudiness. *J. Clim.* **26**, 7507–7524 (2013).

7. S. Bony, J. L. Dufresne, H. Le Treut, J. J. Morcrette, C. Senior, On dynamic and thermodynamic components of cloud changes. *Clim. Dyn.* **22**, 71–86 (2004).

8. J. R. Brown, M. Lengaigne, B. R. Lintner, M. J. Widlansky, K. van der Wiel, C. Dutheil, B. K. Linsley, A. J. Matthews, J. Renwick, South Pacific Convergence Zone dynamics, variability and impacts in a changing climate. *Nat. Rev. Earth Environ.* **1**, 530–543 (2020).

9. A. Muhlbauer, T. P. Ackerman, J. M. Comstock, G. S. Diskin, S. M. Evans, R. P. Lawson, R. T. Marchand, Impact of large-scale dynamics on the microphysical properties of midlatitude cirrus. *J. Geophys. Res.* **119**, 3976–3996 (2014).

10. B. A. Albrecht, Aerosols, cloud microphysics, and fractional cloudiness. *Science* **245**, 1227–1230 (1989).

11. H. Hotta, K. Suzuki, D. Goto, M. Lebsock, Climate impact of cloud water inhomogeneity through microphysical processes in a global climate model. *J. Clim.* **33**, 5195–5212 (2020).

12. O. Boucher, D. Randall, P. Artaxo, C. S. Bretherton, "Clouds and aerosols," in *Climate Change 2013 the Physical Science Basis: Working Group I Contribution to the Fifth Assessment Report of the Intergovernmental Panel on Climate Change* (Cambridge Univ. Press, 2013), pp. 571–657.

13. R. Wood, M. Wyant, C. S. Bretherton, J. Rémillard, P. Kollias, J. Fletcher, J. Stemmler, S. De Szoeke, S. Yuter, M. Miller, D. Mechem, G. Tselioudis, J. C. Chiu, J. A. L. Mann, E. J. O'Connor, R. J. Hogan, X. Dong, M. Miller, V. Ghate, A. Jefferson, Q. Min, P. Minnis, R. Palikonda, B. Albrecht, E. Luke, C. Hannay, Y. Lin, Clouds, aerosols, and precipitation in the marine boundary layer: An arm mobile facility deployment. *Bull. Am. Meteorol. Soc.* **96**, 419–440 (2015).

14. M. D. Zelinka, T. A. Myers, D. T. McCoy, S. Po-Chedley, P. M. Caldwell, P. Ceppi, S. A. Klein, K. E. Taylor, Causes of higher climate sensitivity in CMIP6 models. *Geophys. Res. Lett.* **47**, e2019GL085782 (2020).

15. J. L. Dufresne, S. Bony, An assessment of the primary sources of spread of global warming estimates from coupled atmosphere-ocean models. *J. Clim.* **21**, 5135–5144 (2008).

16. S. C. Sherwood, M. J. Webb, J. D. Annan, K. C. Armour, P. M. Forster, J. C. Hargreaves, G. Hegerl, S. A. Klein, K. D. Marvel, E. J. Rohling, M. Watanabe, T. Andrews, P. Braconnot, C. S. Bretherton, G. L. Foster, Z. Hausfather, A. S. von der Heydt, R. Knutti, T. Mauritsen, J. R. Norris, C. Proistosescu, M. Rugenstein, G. A. Schmidt, K. B. Tokarska, M. D. Zelinka, An assessment of Earth's climate sensitivity using multiple lines of evidence. *Rev. Geophys.* **58**, e2019RG000678 (2020).

17. National Research Council, *Carbon Dioxide and Climate: A Scientific Assessment* (The National Academies Press, 1979).

18. R. T. Wetherald, S. Manabe, Cloud feedback processes in a general circulation model. *J. Atmos. Sci.* **45**, 1397–1416 (1988).

19. J. Hansen, A. Lacis, D. Rind, G. Russell, P. Stone, I. Fung, R. Ruedy, J. Lerner, "Climate sensitivity: Analysis of feedback mechanisms" in *Climate Processes and Climate Sensitivity*, J. E. Hansen, T. Takahashi, Eds. (American Geophysical Union, 1984), pp. 130–163.

20. F. Lehner, C. Deser, N. Maher, J. Marotzke, E. M. Fischer, L. Brunner, R. Knutti, E. Hawkins, Partitioning climate projection uncertainty with multiple large ensembles and CMIP5/6. *Earth Syst. Dyn.* **11**, 491–508 (2020).

21. R. Wood, C. S. Bretherton, D. L. Hartmann, Diurnal cycle of liquid water path over the subtropical and tropical oceans. *Geophys. Res. Lett.* **29**, 7-1–7-4 (2002).

22. C. Deser, C. A. Smith, Diurnal and semidiurnal variations of the surface wind field over the tropical Pacific Ocean. *J. Clim.* **11**, 1730–1748 (1998).

23. J. H. Ruppert Jr., C. Hohenegger, Diurnal circulation adjustment and organized deep convection. *J. Clim.* **31**, 4899–4916 (2018).

24. W. M. Gray, R. W. Jacobson Jr., Diurnal variation of deep cumulus convection. *Mon. Weather Rev.* **105**, 1171–1188 (1977).

25. J. Song, F. Song, Z. Feng, L. R. Leung, C. Li, L. Wu, Realistic precipitation diurnal cycle in global convection-permitting models by resolving mesoscale convective systems. *Geophys. Res. Lett.* **51**, e2024GL109945 (2024).

26. N. G. Loeb, R. Davies, Observational evidence of plane parallel model biases: Apparent dependence of cloud optical depth on solar zenith angle. *J. Geophys. Res. Atmos.* **101**, 1621–1634 (1996).

27. M. J. Webb, A. P. Lock, A. Bodas-Salcedo, S. Bony, J. N. S. Cole, T. Koshiro, H. Kawai, C. Lacagnina, F. M. Selten, R. Roehrig, B. Stevens, The diurnal cycle of marine cloud feedback in climate models. *Clim. Dyn.* **44**, 1419–1436 (2015).

28. K. M. Smalley, M. D. Lebsock, R. Eastman, Diurnal patterns in the observed cloud liquid water path response to droplet number perturbations. *Geophys. Res. Lett.* **51**, e2023GL107323 (2024).

29. G. S. Elsaesser, C. W. O'Dell, M. D. Lebsock, R. Bennartz, T. J. Greenwald, F. J. Wentz, The Multisensor Advanced Climatology of Liquid Water Path (MAC-LWP). *J. Clim.* **30**, 10193–10210 (2017).

30. V. Eyring, S. Bony, G. A. Meehl, C. A. Senior, B. Stevens, R. J. Stouffer, K. E. Taylor, Overview of the Coupled Model Intercomparison Project Phase 6 (CMIP6) experimental design and organization. *Geosci. Model Dev.* **9**, 1937–1958 (2016).

31. J. M. Nugent, H. Brown, A. Kirby, D. T. McCoy, G. Allen, T. Aerenson, S. Burrows, D. Caulton, J. Fan, Y. Feng, A. Gettelman, J. Griswold, D. Jones, L. R. Leung, N. Mahfouz, A. Mikkelsen, J. Muelmenstaedt, Y. Qian, Y. Shan, J. Shpund, I. Silber, C. Song, X. Song, H. Wang, M. Wu, X. Sie, M. Zelinka, D. Zhang, G. J. Zhang, K. Zhang, Overview of the Nephele Perturbed Parameter Ensemble for aerosol-cloud interactions in E3SMv3 [Preprint] (2025); https://doi.org/10.22541/essoar.174907165.57104591/v1.

32. NASA/LARC/SD/ASDC, CERES Energy Balanced and Filled (EBAF) TOA and Surface Monthly means data in netCDF Edition 4.2 [Dataset]. https://doi.org/10.5067/TERRA-AQUA-NOAA20/CERES/EBAF_L3B004.2.

33. N. G. Loeb, S. Kato, K. Loukachine, N. Manalo-Smith, D. R. Doelling, Angular distribution models for top-of-atmosphere radiative flux estimation from the clouds and the Earth's radiant energy system instrument on the *Terra* satellite. Part II: Validation. *J. Atmos. Oceanic Tech.* **24**, 564–584 (2007).

34. N. G. Loeb, S. Kato, K. Loukachine, N. Manalo-Smith, Angular distribution models for top-of-atmosphere radiative flux estimation from the clouds and the Earth's radiant energy system instrument on the *Terra* satellite. Part I: Methodology. *J. Atmos. Oceanic Tech.* **22**, 338–351 (2005).

35. S. Twomey, H. Jacobowitz, H. B. Howell, Light scattering by cloud layers. *J. Atmos. Sci.* **24**, 70–79 (1967).

36. O. Gutjahr, D. Putrasahan, K. Lohmann, J. H. Jungclaus, J. S. Von Storch, N. Brüggemann, H. Haak, A. Stössel, Max Planck Institute Earth System Model (MPI-ESM1.2) for the High-Resolution Model Intercomparison Project (HighResMIP). *Geosci. Model Dev.* **12**, 3241–3281 (2019).

37. N. C. Swart, J. N. S. Cole, V. V. Kharin, M. Lazare, J. F. Scinocca, N. P. Gillett, J. Anstey, V. Arora, J. R. Christian, S. Hanna, Y. Jiao, W. G. Lee, F. Majaess, O. A. Saenko, C. Seiler, C. Seinen, A. Shao, M. Sigmond, L. Solheim, K. Von Salzen, D. Yang, B. Winter, The Canadian Earth System Model version 5 (CanESM5.0.3). *Geosci. Model Dev.* **12**, 4823–4873 (2019).

38. W. L. Gates, J. S. Boyle, C. Covey, C. G. Dease, C. M. Doutriaux, R. S. Drach, M. Fiorino, P. J. Gleckler, J. J. Hnilo, S. M. Marlais, T. J. Phillips, G. L. Potter, B. D. Santer, K. R. Sperber, K. E. Taylor, D. N. Williams, An overview of the results of the Atmospheric Model Intercomparison Project (AMIP I). *Bull. Amer. Meteor. Soc.* **80**, 29–56 (1999).

39. R. Wood, C. S. Bretherton, On the relationship between stratiform low cloud cover and lower-tropospheric stability. *J. Clim.* **19**, 6425–6432 (2006).

40. S. A. Klein, D. L. Hartmann, The seasonal cycle of low stratiform clouds. *J. Clim.* **6**, 1587–1606 (1993).

41. D. T. McCoy, M. E. Frazer, J. Mülmenstädt, I. Tan, C. R. Terai, M. D. Zelinka, "Extratropical cloud feedbacks" in *Clouds and Their Climatic Impacts*, S. C. Sullivan, C. Hoose, Eds. (American Geophysical Union, 2023).

42. E3SM Project, DOE, Energy Exascale Earth System Model v3.0.1. (2024); https://doi.org/10.11578/E3SM/dc.20240930.1.

43. S. W. Nesbitt, E. J. Zipser, The diurnal cycle of rainfall and convective intensity according to three years of TRMM measurements. *J. Clim.* **16**, 1456–1475 (2003).

44. S. W. Nesbitt, E. J. Zipser, D. J. Cecil, A census of precipitation features in the tropics using TRMM: Radar, ice scattering, and lightning observations. *J. Clim.* **13**, 4087–4106 (2000).

45. R. Wood, Stratocumulus clouds. *Mon. Weather Rev.* **140**, 2373–2423 (2012).

46. Y. F. Ma, J. G. Pedersen, W. W. Grabowski, M. K. Kopec, S. P. Malinowski, Influences of subsidence and free-tropospheric conditions on the nocturnal growth of nonclassical marine stratocumulus. *J. Adv. Model. Earth Syst.* **10**, 2706–2730 (2018).

47. G. V. Cesana, A. S. Ackerman, N. Črnivec, R. Pincus, H. Chepfer, An observation-based method to assess tropical stratocumulus and shallow cumulus clouds and feedbacks in CMIP6 and CMIP5 models. *Environ. Res. Commun.* **5**, 045001 (2023).

48. F. Brient, T. Schneider, Z. Tan, S. Bony, X. Qu, A. Hall, Shallowness of tropical low clouds as a predictor of climate models' response to warming. *Clim. Dyn.* **47**, 433–449 (2016).

49. S. C. Sherwood, S. Bony, J. L. Dufresne, Spread in model climate sensitivity traced to atmospheric convective mixing. *Nature* **505**, 37–42 (2014).

50. C. S. Bretherton, Insights into low-latitude cloud feedbacks from high-resolution models. *Philos. Trans. R. Soc. A* **373**, 20140415 (2015).

51. M. Zhang, C. S. Bretherton, P. N. Blossey, P. H. Austin, J. T. Bacmeister, S. Bony, F. Brient, S. K. Cheedela, A. Cheng, A. D. Del Genio, S. R. De Roode, S. Endo, C. N. Franklin, J. C. Golaz, C. Hannay, T. Heus, F. A. Isotta, J. L. Dufresne, I. S. Kang, H. Kawai, M. Köhler, V. E. Larson, Y. Liu, A. P. Lock, U. Lohmann, M. F. Khairoutdinov, A. M. Molod, R. A. J. Neggers, P. Rasch, I. Sandu, R. Senkbeil, A. P. Siebesma, C. Siegenthaler-Le Drian, B. Stevens, M. J. Suarez, K. M. Xu, K. von Salzen, M. J. Webb, A. Wolf, M. Zhao, CGILS: Results from the first phase of an international project to understand the physical mechanisms of low cloud feedbacks in single column models. *J. Adv. Model. Earth Syst.* **5**, 826–842 (2013).

52. C. Nam, S. Bony, J.-L. Dufresne, H. Chepfer, The 'too few, too bright' tropical low-cloud problem in CMIP5 models. *Geophys. Res. Lett.* **39**, L21801 (2012).

53. D. Konsta, J. L. Dufresne, H. Chepfer, J. Vial, T. Koshiro, H. Kawai, A. Bodas-Salcedo, R. Roehrig, M. Watanabe, T. Ogura, Low-level marine tropical clouds in six CMIP6 models are too few, too bright but also too compact and too homogeneous. *Geophys. Res. Lett.* **49**, e2021GL097593 (2022).

54. R. Eastman, S. G. Warren, Diurnal cycles of cumulus, cumulonimbus, stratus, stratocumulus, and fog from surface observations over land and ocean. *J. Clim.* **27**, 2386–2404 (2014).

55. J. Latham, K. Bower, T. Choularton, H. Coe, P. Connolly, G. Cooper, T. Craft, J. Foster, A. Gadian, L. Galbraith, H. Iacovides, D. Johnston, B. Launder, B. Leslie, J. Meyer, A. Neukermans, B. Ormond, B. Parkes, P. Rasch, J. Rush, S. Salter, T. Stevenson, H. Wang, Q. Wang, R. Wood, Marine cloud brightening. *Philos. Trans. R. Soc. A Math. Phys. Eng. Sci.* **370**, 4217–4262 (2012).

56. A. K. L. Jenkins, P. M. Forster, L. S. Jackson, The effects of timing and rate of marine cloud brightening aerosol injection on albedo changes during the diurnal cycle of marine stratocumulus clouds. *Atmos. Chem. Phys.* **13**, 1659–1673 (2013).

57. C. W. Stjern, H. Muri, L. Ahlm, O. Boucher, J. N. S. Cole, D. Ji, A. Jones, J. Haywood, B. Kravitz, A. Lenton, J. C. Moore, U. Niemeier, S. J. Phipps, H. Schmidt, S. Watanabe, J. E. Kristjánsson, Response to marine cloud brightening in a multi-model ensemble. *Atmos. Chem. Phys.* **18**, 621–634 (2018).

58. T. Aerenson, R. Marchand, How do differences in the simulation of present-day clouds affect cloud feedbacks? *J. Geophys. Res. Atmos.* **130**, e2025JD044020 (2025).

59. C. S. Bretherton, P. M. Caldwell, Combining emergent constraints for climate sensitivity. *J. Climate* **33**, 7413–7430 (2020).

60. F. Brient, Reducing uncertainties in climate projections with emergent constraints: Concepts, examples and prospects. *Adv. Atmos. Sci.* **37**, 1–15 (2020).

61. D. P. Grosvenor, O. Sourdeval, R. Wood, Parameterizing cloud top effective radii from satellite retrieved values, accounting for vertical photon transport: Quantification and correction of the

resulting bias in droplet concentration and liquid water path retrievals. *Atmos. Meas. Tech.* **11**, 4273–4289 (2018).

62. J. E. Kay, B. R. Hillman, S. A. Klein, Y. Zhang, B. Medeiros, R. Pincus, A. Gettelman, B. Eaton, J. Boyle, R. Marchand, T. P. Ackerman, Exposing global cloud biases in the Community Atmosphere Model (CAM) using satellite observations and their corresponding instrument simulators. *J. Clim.* **25**, 5190–5207 (2012).

63. R. C. Scott, T. A. Myers, J. R. Norris, M. D. Zelinka, S. A. Klein, M. Sun, D. R. Doelling, Observed sensitivity of low-cloud radiative effects to meteorological perturbations over the global oceans. *J. Clim.* **33**, 7717–7734 (2020).

64. I. Tan, T. Storelvmo, M. D. Zelinka, Observational constraints on mixed-phase clouds imply higher climate sensitivity. *Science* **352**, 224–227 (2016).

65. C. W. Thackeray, M. D. Zelinka, J. Norris, A. Hall, S. Po-Chedley, Relationship between tropical cloud feedback and climatological bias in clouds. *Geophys. Res. Lett.* **51**, e2024GL111347 (2024).

66. L. A. Lee, K. S. Carslaw, K. J. Pringle, G. W. Mann, D. V. Spracklen, Emulation of a complex global aerosol model to quantify sensitivity to uncertain parameters. *Atmos. Chem. Phys.* **11**, 12253–12273 (2011).

67. R. Marchand, T. Ackerman, M. Smyth, W. B. Rossow, A review of cloud top height and optical depth histograms from MISR, ISCCP, and MODIS. *J. Geophys. Res. Atmos.* **115**, D16206 (2010).

68. T. Wu, Y. Lu, Y. Fang, X. Xin, L. Li, W. Li, W. Jie, J. Zhang, Y. Liu, L. Zhang, F. Zhang, Y. Zhang, F. Wu, J. Li, M. Chu, Z. Wang, X. Shi, X. Liu, M. Wei, A. Huang, Y. Zhang, X. Liu, The Beijing Climate Center Climate System Model (BCC-CSM): The main progress from CMIP5 to CMIP6. *Geosci. Model Dev.* **12**, 1573–1600 (2019).

69. T. Wu, F. Zhang, J. Zhang, W. Jie, Y. Zhang, F. Wu, L. Li, J. Yan, X. Liu, X. Lu, H. Tan, L. Zhang, J. Wang, A. Hu, Beijing Climate Center Earth System Model version 1 (BCC-ESM1):

Model description and evaluation of aerosol simulations. *Geosci. Model Dev.* **13**, 977–1005 (2020).

70. X. Rong, J. Li, H. Chen, Y. Xin, J. Su, L. Hua, T. Zhou, Y. Qi, Z. Zhang, G. Zhang, J. Li, The CAMS climate system model and a basic evaluation of its climatology and climate variability simulation. *J. Meteorol. Res.* **32**, 839–861 (2018).

71. G. Danabasoglu, J. F. Lamarque, J. Bacmeister, D. A. Bailey, A. K. DuVivier, J. Edwards, L. K. Emmons, J. Fasullo, R. Garcia, A. Gettelman, C. Hannay, M. M. Holland, W. G. Large, P. H. Lauritzen, D. M. Lawrence, J. T. M. Lenaerts, K. Lindsay, W. H. Lipscomb, M. J. Mills, R. Neale, K. W. Oleson, B. Otto-Bliesner, A. S. Phillips, W. Sacks, S. Tilmes, L. van Kampenhout, M. Vertenstein, A. Bertini, J. Dennis, C. Deser, C. Fischer, B. Fox-Kemper, J. E. Kay, D. Kinnison, P. J. Kushner, V. E. Larson, M. C. Long, S. Mickelson, J. K. Moore, E. Nienhouse, L. Polvani, P. J. Rasch, W. G. Strand, The Community Earth System Model Version 2 (CESM2). *J. Adv. Model. Earth Syst.* **12**, e2019MS001916 (2020).

72. A. Voldoire, D. Saint-Martin, S. Sénési, B. Decharme, A. Alias, M. Chevallier, J. Colin, J. F. Guérémy, M. Michou, M. P. Moine, P. Nabat, R. Roehrig, D. Salas y Mélia, R. Séférian, S. Valcke, I. Beau, S. Belamari, S. Berthet, C. Cassou, J. Cattiaux, J. Deshayes, H. Douville, C. Ethé, L. Franchistéguy, O. Geoffroy, C. Lévy, G. Madec, Y. Meurdesoif, R. Msadek, A. Ribes, E. Sanchez-Gomez, L. Terray, R. Waldman, Evaluation of CMIP6 DECK experiments with CNRM-CM6-1. *J. Adv. Model. Earth Syst.* **11**, 2177–2213 (2019).

73. R. Séférian, P. Nabat, M. Michou, D. Saint-Martin, A. Voldoire, J. Colin, B. Decharme, C. Delire, S. Berthet, M. Chevallier, S. Sénési, L. Franchisteguy, J. Vial, M. Mallet, E. Joetzjer, O. Geoffroy, J. F. Guérémy, M. P. Moine, R. Msadek, A. Ribes, M. Rocher, R. Roehrig, D. Salas-y-Mélia, E. Sanchez, L. Terray, S. Valcke, R. Waldman, O. Aumont, L. Bopp, J. Deshayes, C. Éthé, G. Madec, Evaluation of CNRM Earth System Model, CNRM-ESM2-1: Role of Earth system processes in present-day and future climate. *J. Adv. Model. Earth Syst.* **11**, 4182–4227 (2019).

74. J.-C. Golaz, P. M. Caldwell, L. P. Van Roekel, M. R. Petersen, Q. Tang, J. D. Wolfe, G. Abeshu, V. Anantharaj, X. S. Asay-Davis, D. C. Bader, S. A. Baldwin, G. Bisht, P. A.

Bogenschutz, M. Branstetter, M. A. Brunke, S. R. Brus, S. M. Burrows, P. J. Cameron-Smith, A. S. Donahue, M. Deakin, R. C. Easter, K. J. Evans, Y. Feng, M. Flanner, J. G. Foucar, J. G. Fyke, B. M. Griffin, C. Hannay, B. E. Harrop, M. J. Hoffman, E. C. Hunke, R. L. Jacob, D. W. Jacobsen, N. Jeffery, P. W. Jones, N. D. Keen, S. A. Klein, V. E. Larson, L. R. Leung, H. Y. Li, W. Lin, W. H. Lipscomb, P. L. Ma, S. Mahajan, M. E. Maltrud, A. Mametjanov, J. L. McClean, R. B. McCoy, R. B. Neale, S. F. Price, Y. Qian, P. J. Rasch, J. E. J. Reeves Eyre, W. J. Riley, T. D. Ringler, A. F. Roberts, E. L. Roesler, A. G. Salinger, Z. Shaheen, X. Shi, B. Singh, J. Tang, M. A. Taylor, P. E. Thornton, A. K. Turner, M. Veneziani, H. Wan, H. Wang, S. Wang, D. N. Williams, P. J. Wolfram, P. H. Worley, S. Xie, Y. Yang, J. H. Yoon, M. D. Zelinka, C. S. Zender, X. Zeng, C. Zhang, K. Zhang, Y. Zhang, X. Zheng, T. Zhou, Q. Zhu, The DOE E3SM Coupled Model Version 1: Overview and evaluation at standard resolution. *J. Adv. Model. Earth Syst.* **11**, 2089–2129 (2019).

75. R. Döscher, M. Acosta, A. Alessandri, P. Anthoni, T. Arsouze, T. Bergman, R. Bernardello, S. Boussetta, L. P. Caron, G. Carver, M. Castrillo, F. Catalano, I. Cvijanovic, P. Davini, E. Dekker, F. J. Doblas-Reyes, D. Docquier, P. Echevarria, U. Fladrich, R. Fuentes-Franco, M. Gröger, J. V. Hardenberg, J. Hieronymus, M. P. Karami, J. P. Keskinen, T. Koenigk, R. Makkonen, F. Massonnet, M. Ménégoz, P. A. Miller, E. Moreno-Chamarro, L. Nieradzik, T. Van Noije, P. Nolan, D. O’Donnell, P. Ollinaho, G. van den Oord, P. Ortega, O. T. Prims, A. Ramos, T. Reerink, C. Rousset, Y. Ruprich-Robert, P. L. Sager, T. Schmith, R. Schrödner, F. Serva, V. Sicardi, M. S. Madsen, B. Smith, T. Tian, E. Tourigny, P. Uotila, M. Vancoppenolle, S. Wang, D. Wårlind, U. Willén, K. Wyser, S. Yang, X. Yepes-Arbós, Q. Zhang, The EC-Earth3 Earth system model for the coupled model intercomparison project 6. *Geosci. Model Dev.* **15**, 2973–3020 (2022).

76. M. Kelley, G. A. Schmidt, L. S. Nazarenko, S. E. Bauer, R. Ruedy, G. L. Russell, A. S. Ackerman, I. Aleinov, M. Bauer, R. Bleck, V. Canuto, G. Cesana, Y. Cheng, T. L. Clune, B. I. Cook, C. A. Cruz, A. D. Del Genio, G. S. Elsaesser, G. Faluvegi, N. Y. Kiang, D. Kim, A. A. Lacis, A. Leboissetier, A. N. LeGrande, K. K. Lo, J. Marshall, E. E. Matthews, S. McDermid, K. Mezuman, R. L. Miller, L. T. Murray, V. Oinas, C. Orbe, C. P. García-Pando, J. P. Perlwitz, M. J. Puma, D. Rind, A. Romanou, D. T. Shindell, S. Sun, N. Tausnev, K. Tsigaridis, G. Tselioudis,

E. Weng, J. Wu, M. S. Yao, GISS-E2.1: Configurations and climatology. *J. Adv. Model. Earth Syst.* **12**, e2019MS002025 (2020).

77. M. J. Roberts, A. Baker, E. W. Blockley, D. Calvert, A. Coward, H. T. Hewitt, L. C. Jackson, T. Kuhlbrodt, P. Mathiot, C. D. Roberts, R. Schiemann, J. Seddon, B. Vannière, P. Luigi Vidale, Description of the resolution hierarchy of the global coupled HadGEM3-GC3.1 model as used in CMIP6 HighResMIP experiments. *Geosci. Model Dev.* **12**, 4999–5028 (2019).

78. O. Boucher, J. Servonnat, A. L. Albright, O. Aumont, Y. Balkanski, V. Bastrikov, S. Bekki, R. Bonnet, S. Bony, L. Bopp, P. Braconnot, P. Brockmann, P. Cadule, A. Caubel, F. Cheruy, F. Codron, A. Cozic, D. Cugnet, F. D'Andrea, P. Davini, C. de Lavergne, S. Denvil, J. Deshayes, M. Devilliers, A. Ducharne, J. L. Dufresne, E. Dupont, C. Éthé, L. Fairhead, L. Falletti, S. Flavoni, M. A. Foujols, S. Gardoll, G. Gastineau, J. Ghattas, J.-Y. Grandpeix, B. Guenet, Lionel, E. Guez, E. Guilyardi, M. Guimberteau, D. Hauglustaine, F. Hourdin, A. Idelkadi, S. Joussaume, M. Kageyama, M. Khodri, G. Krinner, N. Lebas, G. Levavasseur, C. Lévy, L. Li, F. Lott, T. Lurton, S. Luyssaert, G. Madec, J. B. Madeleine, F. Maignan, M. Marchand, O. Marti, L. Mellul, Y. Meurdesoif, J. Mignot, I. Musat, C. Ottlé, P. Peylin, Y. Planton, J. Polcher, C. Rio, N. Rochetin, C. Rousset, P. Sepulchre, A. Sima, D. Swingedouw, R. Thiéblemont, A. K. Traore, M. Vancoppenolle, J. Vial, J. Vialard, N. Viovy, N. Vuichard, Presentation and evaluation of the IPSL-CM6A-LR climate model. *J. Adv. Model. Earth Syst.* **12**, e2019MS002010 (2020).

79. T. Hajima, M. Watanabe, A. Yamamoto, H. Tatebe, M. A. Noguchi, M. Abe, R. Ohgaito, A. Ito, D. Yamazaki, H. Okajima, A. Ito, K. Takata, K. Ogochi, S. Watanabe, M. Kawamiya, Development of the MIROC-ES2L Earth system model and the evaluation of biogeochemical processes and feedbacks. *Geosci. Model Dev.* **13**, 2197–2244 (2020).

80. H. Tatebe, T. Ogura, T. Nitta, Y. Komuro, K. Ogochi, T. Takemura, K. Sudo, M. Sekiguchi, M. Abe, F. Saito, M. Chikira, S. Watanabe, M. Mori, N. Hirota, Y. Kawatani, T. Mochizuki, K. Yoshimura, K. Takata, R. O'Ishi, D. Yamazaki, T. Suzuki, M. Kurogi, T. Kataoka, M. Watanabe, M. Kimoto, Description and basic evaluation of simulated mean state, internal variability, and climate sensitivity in MIROC6. *Geosci. Model Dev.* **12**, 2727–2765 (2019).

81. S. Yukimoto, H. Kawai, T. Koshiro, N. Oshima, K. Yoshida, S. Urakawa, H. Tsujino, M. Deushi, T. Tanaka, M. Hosaka, S. Yabu, H. Yoshimura, E. Shindo, R. Mizuta, A. Obata, Y. Adachi, M. Ishii, The meteorological research institute Earth system model version 2.0, MRI-ESM2.0: Description and basic evaluation of the physical component. *J. Meteorol. Soc. Jpn* **97**, 931–965 (2019).

82. J. Cao, B. Wang, Y. M. Yang, L. Ma, J. Li, B. Sun, Y. Bao, J. He, X. Zhou, L. Wu, The NUIST Earth System Model (NESM) version 3: Description and preliminary evaluation. *Geosci. Model Dev.* **11**, 2975–2993 (2018).

83. S. Park, J. Shin, S. Kim, E. Oh, Y. Kim, Global climate simulated by the Seoul National University Atmosphere Model Version 0 with a Unified Convection Scheme (SAM0-UNICON). *J. Clim.* **32**, 2917–2949 (2019).

84. A. A. Sellar, C. G. Jones, J. P. Mulcahy, Y. Tang, A. Yool, A. Wiltshire, F. M. O'Connor, M. Stringer, R. Hill, J. Palmieri, S. Woodward, L. de Mora, T. Kuhlbrodt, S. T. Rumbold, D. I. Kelley, R. Ellis, C. E. Johnson, J. Walton, N. L. Abraham, M. B. Andrews, T. Andrews, A. T. Archibald, S. Berthou, E. Burke, E. Blockley, K. Carslaw, M. Dalvi, J. Edwards, G. A. Folberth, N. Gedney, P. T. Griffiths, A. B. Harper, M. A. Hendry, A. J. Hewitt, B. Johnson, A. Jones, C. D. Jones, J. Keeble, S. Liddicoat, O. Morgenstern, R. J. Parker, V. Predoi, E. Robertson, A. Siahaan, R. S. Smith, R. Swaminathan, M. T. Woodhouse, G. Zeng, M. Zerroukat, UKESM1: Description and evaluation of the U.K. Earth System Model. *J. Adv. Model. Earth Syst.* **11**, 4513–4558 (2019).

85. C. S. Bretherton, M. Widmann, V. P. Dymnikov, J. M. Wallace, I. Bladé, The effective number of spatial degrees of freedom of a time-varying field. *J. Clim.* **12**, 1990–2009 (1999).
